# Transcriptome Profiling of the Theca Interna from Bovine Ovarian Follicles during Atresia

**DOI:** 10.1371/journal.pone.0099706

**Published:** 2014-06-23

**Authors:** Nicholas Hatzirodos, Helen F. Irving-Rodgers, Katja Hummitzsch, Raymond J. Rodgers

**Affiliations:** Research Centre for Reproductive Health, Discipline of Obstetrics and Gynaecology, School of Paediatrics and Reproductive Health, Robinson Research Institute, University of Adelaide, Adelaide, SA, Australia; University of Nevada School of Medicine, United States of America

## Abstract

The theca interna is a specialized stromal layer that envelops each growing ovarian follicle. It contains capillaries, fibroblasts, immune cells and the steroidogenic cells that synthesize androgens for conversion to estradiol by the neighboring granulosa cells. During reproductive life only a small number of follicles will grow to a sufficient size to ovulate, whereas the majority of follicles will undergo regression/atresia and phagocytosis by macrophages. To identify genes which are differentially regulated in the theca interna during follicular atresia, we undertook transcriptome profiling of the theca interna from healthy (n = 10) and antral atretic (n = 5) bovine follicles at early antral stages (<5 mm). Principal Component Analyses and hierarchical classification of the signal intensity plots for the arrays showed primary clustering into two groups, healthy and atretic. A total of 543 probe sets were differentially expressed between the atretic and healthy theca interna. Further analyses of these genes by Ingenuity Pathway Analysis and Gene Ontology Enrichment Analysis Toolkit software found most of the genes being expressed were related to cytokines, hormones and receptors as well as the cell cycle and DNA replication. Cell cycle genes which encode components of the replicating chromosome complex and mitotic spindle were down-regulated in atretic theca interna, whereas stress response and inflammation-related genes such as *TP53*, *IKBKB* and *TGFB1* were up-regulated. In addition to cell cycle regulators, upstream regulators that were predicted to be inhibited included Retinoblastoma 1, E2 transcription factor 1, and hepatocyte growth factor. Our study suggests that during antral atresia of small follicles in the theca interna, arrest of cell cycle and DNA replication occurs rather than up- regulation of apoptosis-associated genes as occurs in granulosa cells.

## Introduction

Mature ovarian follicles consist of an oocyte surrounded by epithelial granulosa cells all enclosed by a basal lamina which separates these cells from the surrounding stromal thecal layers. The thecal layers can be divided into the theca interna, which is closest to the follicular basal lamina and contains steroidogenic cells, fibroblastic cells, immune cells and capillaries, and the theca externa which is composed mostly of fibroblastic cells and smooth muscle-like cells. The main function of the theca interna is to produce androgens which serve as precursors for estradiol synthesis by the granulosa cells [1]. It also supplies nutrients and growth factors to the follicle via its vasculature. Furthermore, the follicular fluid which fills the antrum of mature ovarian follicles, originates from fluid transported in the capillaries of the theca interna [2].

In bovine ovaries two morphological phenotypes of healthy follicles have been observed at sizes <5 mm [3]. These differ in the structure of the follicular basal lamina and the shape of the basally-situated granulosa cells. The aligned/rounded (basal lamina phenotype/shape of basal granulosa cell) phenotype differs from the loopy/rounded phenotype in the quality of their oocytes and these follicle phenotypes have also been observed in human ovaries [4]. The loopy basal lamina was so named as its phenotype was composed of additional layers or loops which branched from the innermost basal lamina layer and the basal granulosa cells were columnar in shape [3]. The origin of these two phenotypes is predicted to be due to differential rates of follicular antrum expansion, with the aligned/rounded phenotype being faster than the other [5].

During each cycle the majority of growing follicles undergo follicular atresia potentially as a means to limit the numbers of follicles that ovulate and to assist in co-ordination of the timing of ovulation. During atresia, cell death and phagocytosis by macrophages is observed [5]. For bovine follicles <5 mm, two types of atresia have been described based on the initial location of apoptotic nuclei in the granulosa cell layers, either basally or antrally situated [5], [6]. Most of the studies on follicular atresia have focused on granulosa cells and oocytes, but less is known about the thecal cells. A recent study by Christenson et al [7] surveyed the transcriptome of the theca interna during development under the influence of the LH surge and identified novel genes associated with this process, however, the transcriptome in the theca during atresia has not been investigated. There is evidence that the theca interna may play a role in follicular atresia through elevated expression of fibroblast growth factor 18 (FGF18). Addition of FGF18 to granulosa cells *in vitro*, led to a decline in steroidogenesis as well as cell cycle progression and increased fragmentation of granulosa cell DNA [8].

Basal atresia affects the granulosa cells closest to the basal lamina and can lead to disruptions in the basal lamina and entry of macrophages, fibroblasts and endothelial cells into the damaged granulosa cell layers [9]. The surviving granulosa cells near the antrum increase expression of the steroidogenic enzymes cholesterol side-chain cleavage cytochrome P450 and 3β-hydroxysteroid dehydrogenase, leading to higher progesterone concentration in the follicular fluid of these atretic follicles [10]. Androstenedione and testosterone concentrations in the follicular fluid are reduced in basal atresia compared to healthy and antral atretic follicles [10]. This is presumably due to increased apoptosis of thecal steroidogenic cells in basal atretic follicles [11]. Insulin-like growth factor binding proteins 2, 4 and 5 are increased [12] in follicles of both atretic types compared with healthy follicles.

In contrast to basal atresia, during antral atresia the granulosa cells closest to the follicular antrum undergo cell death first, whereas the cells close to the basal lamina and the basal lamina itself remain intact until later in the process [11]. Also in contrast to basal atretic follicles, the thecal layers in follicles undergoing antral atresia appear largely unchanged compared to healthy follicles [11], but it cannot be excluded that changes at the gene level occur in thecal cells of this atretic type. Therefore in the current study we examined the theca interna of the small healthy follicles of both the aligned/rounded and loopy/columnar phenotype [3] and small atretic follicles of the antral atretic phenotype [6] where less is known about potential changes in the theca interna [11].

## Materials and Methods

### Selection of bovine ovarian follicles

Pairs of ovaries were collected from non-pregnant cycling *Bos taurus* heifers at an abattoir (T&R Pastoral, Murray Bridge, SA, Australia). Follicles with an external diameter of 3–5 mm as measured by callipers were dissected for classification and analysis. Granulosa cells were aspirated and scraped from each follicle with a Pasteur pipette, whose tip had been blunted previously by heating, and the granulosa cells were discarded. The theca interna was then dissected from the follicle wall under a Zeiss Stemi D4 stereomicroscope (Zeiss Pty Ltd., North Ryde, NSW, Australia) in cold Hank's balanced-salt solution with Mg^2+^ and Ca^2+^ (Sigma-Aldrich, Castle Hill, NSW, Australia) and stored at −80°C prior to RNA extraction. An excised portion of the follicle wall (2×2×2 mm) was taken prior to granulosa and thecal cell removal and fixed in 2.5% glutaraldehyde in 0.1M phosphate buffer for subsequent histological assessment. Follicles were classified as healthy or atretic based upon the morphology of the membrana granulosa and the presence or absence of apoptotic cells as previously described [3], [6], [12]. Only healthy and antral atretic follicles [6] were examined in this study. A sub-classification of the healthy follicles as aligned/rounded or loopy/columnar was made based on the shape of the granulosa cells in the basal layer adjacent to the follicular basal lamina [3].

### RNA preparation and microarray analyses

RNA was extracted from theca interna by the Trizol method (Life Technologies, Mt Waverley, VIC, Australia). Briefly, each sample was homogenized in 1 ml of Trizol with 1.4 mm ceramic beads in a Precellys 24 Bead Mill Homogenizer (Omni International, Kennesaw, Georgia, USA) with two 10 s cycles of 6,000 rpm each. The samples were then extracted with 200 µl of chloroform and the aqueous phase was purified through a Qiagen RNEasy mini-preparative column (Qiagen, Hilden, Germany) according to the manufacturer's instructions. Five µg of RNA was treated to remove genomic DNA contamination with 2 units of DNAse 1 (Ambion Life Technologies) prior to labeling for microarray analyses. The integrity of all the RNA samples was assessed by microfluidic analyses on a 2000 BioAnalyzer (Agilent, Santa Clara, CA, USA) and were all found to have RNA integrity numbers (RIN)≥8.

DNAse-treated thecal RNA of 100 ng from each individual follicle was labeled using the 3′IVT Express labeling kit (Affymetrix, Santa Clara, CA, USA). In brief, the RNA was reverse transcribed using a T7 oligo dT primer followed by second-strand synthesis. *In vitro* transcription reactions were performed in batches to generate biotinylated cRNA targets, which were subsequently chemically fragmented at 95°C for 35 min. Ten µg of the fragmented biotinylated cRNA was hybridized at 45°C for 16 h to Affymetrix GeneChip Bovine Genome Arrays, which contain 24,128 probe sets representing over 23,000 transcripts and variants, including 19,000 UniGene clusters. The arrays were then washed and stained with streptavidin-phycoerythrin (final concentration 10 µg/ml). Signal amplification was achieved by using a biotinylated anti-streptavidin antibody. The array was then scanned according to the manufacturer's instructions (Affymetrix GeneChip Expression Analysis Technical Manual). The arrays were inspected for defects or artefacts. The array data were converted to CEL file format for analyses.

### Microarray data analyses

Quality control for the cDNA labeling was determined by the use of internal array controls. All the arrays passed these controls. The array data was subjected to Robust Multi Array Average (RMA) summarization [13] and quantile normalization [14] which was considered to be statistically appropriate treatment for normally-distributed data from arrays of this size (greater than 20,000 probe sets).

The 15 arrays were analyzed as part of a larger set of CEL files (which additionally included samples of thecal tissue RNA from 4 large follicles as discussed elsewhere [15]) and were uploaded to the Partek GS software program. Probe sets were filtered such that only those with a log_2_ signal intensity of >3.0 for ≥50% of the arrays of one follicle type were considered to be above the detection threshold. Before statistical analyses, the data were first subjected to PCA [16] and hierarchical clustering analyses to compare the gene expression patterns of the arrays in terms of our classification. Hierarchical clustering was performed using the Euclidian algorithm for dissimilarity with average linkage. The expression data were analyzed by ANOVA using method of moments estimation [17] with post-hoc step-up FDR test for multiple comparisons. The fold change in expression for each gene was based on the non-log transformed values after correction and normalization. The microarray CEL files, normalized data and experimental information have been deposited in the Gene Expression Omnibus (GEO) [18], accessible by this accession number containing the series record (GSE49505).

These differentially expressed genes were uploaded to the Ingenuity database (Ingenuity Systems, Redwood City, CA, USA) for pathway and functional analysis as described previously [19], and were further annotated and classified based on the Gene Ontology (GO) consortium annotations from the GO *Bos taurus* database (2010/02/24) [20] using GOEAST (Gene Ontology Enrichment Analysis Software Toolkit [21]).

### Validation by quantitative real-time PCR

Synthesis of cDNA was performed as previously [22] and briefly described below. Total RNA (200 ng) of theca interna from small healthy and atretic follicles (n = 7 each, from the same samples as used for the microarray, for the atretic group 2 additional samples were included,) was reverse transcribed with SuperScript® III Transcriptase (Life Technologies) using random hexamer primers (Geneworks, Thebarton, SA, Australia) according to the manufacturer's instructions. Two free web-based programs, Primer3Plus [23] and NetPrimer (Premier Biosoft International, Palo Alto, CA), were used to design primers to bovine sequences of all genes shown in Table 1. The real time PCR was performed on a Biomark HD system (Fluidigm Corporation, San Francisco, CA, USA) at the ACRF Cancer Genomics Facility, Adelaide, Australia. The reaction started with a pre-amplification step consisting of a 95°C hold for 10 min, followed by 12 cycles of 95°C for 15 s and 60°C for 4 min each using 50 nM of each primer and 1.25 µl of cDNA in 5 µl. The amplified product was then diluted 1 in 5 and added in 0.05 µl to the final reaction volume of 0.1 µl which contained 500 nM of each primer per assay. The final amplification conditions were a 60 s activation step at 95°C, followed by 30 cycles of 96°C denaturation for 5 s and 60°C annealing/extension for 20 s using SsoFast EvaGreen Supermix With Low ROX (Bio rad, Hercules, Ca, USA) which contains a fluorescent intercalating agent for measuring amplification. The expression values for each gene were determined as the geometric mean of the ratio of 2^−Δ Ct^ for the target gene to *PPIA* and *GAPDH*.

**Table 1:** Primer sequences used for qRT-PCR validation of the microarray data.

Gene name	Gene Symbol	GenBank Accession No.	Primers (5′-3′) (F = forward, R = reverse)	Size (bp)
Glyceraldehyde 3-phosphate dehydrogenase	*GAPDH*	XR_027767	F : ACCACTTTGGCATCGTGGAG	76
			R : GGGCCATCCACAGTCTTCTG	
Peptidylprolyl isomerase A (cyclophilin A)	*PPIA*	NM_178320.2	F : CTGGCATCTTGTCCATGGCAAA	202
			R : CCACAGTCAGCAATGGTGATCTTC	
Glycoprotein (transmembrane) nmb	*GPNMB*	NM_001038065.1	F : CTCGTCACTGTGATCGCCTTT	70
			R : CAGGGCTGTTTTCTATTGGTTTG	
CD68	*CD68*	NM_001045902	F : GGCCTTTGGACCAAGTTTCTC	76
			R : CGAGTAAGATCAGGCCGATGA	
CD36/thrombospondin receptor	*CD36*	NM_001046239.1	F : TCTCTTTCCTGCAGCCCAAT	70
			R : AACGTGTCATCCTCAGTTCCAA	
Adrenomedullin	*ADM*	NM_173888.3	F : GGGTCGCTCGCCTTCCTA	121
			R : AGCTACTGGACTCGCGAAGTTC	
Neurotrophic tyrosine kinase, receptor, type 2	*NTRK2*	NM_001075225.1	F : TGCTGAAGTTGGCAAGACAC	129
			R : CATCAACCAACAAGCACCAC	
Cyclin-dependent kinase inhibitor 1B	*CDKN1B*	NM_001100346.1	F : GAGCAGTGCCCTGGGATAAG	100
			R : GGGAACCGTCTGAAACATTTTC	
Pituitary tumour growth factor	*PTTG1*	NM_001034310.2	F : CCCGCCTCCCCTTGAGT	58
			R : TCAAGCTCCCTCTCCTCATCA	
Cytochrome P450 cholesterol side-chain cleavage	*CYP11A1*	BC133389	F : CACTTTCGCCACATCGAGAA	86
			R : TGAATGATATAAACTGACTCCAAATTGC	
Centromere protein F, 350/400 kDa (mitosin)	*CENPF*	XM_002694283.1	F : CGACATCCCAACCGGAAAG	141
			R : TTGGAGGTCTCGGTGAGATTTT	
Cyclin E2	*CCNE2*	NM_001015665.1	F : CCTCATTATTCATTGCTTCCAAAC	89
			R : TTCACTGCAAGCACCATCAG	

## Results and Discussion

### Statistical analyses of differentially expressed genes

Theca interna from a total of 15 small antral follicles (3–5 mm in diameter, each follicle was from a different animal) were classified on the basis of follicle phenotype as described in the methods and then examined by microarray analyses of gene expression. These consisted of three groups each of n = 5. One group contained antral atretic follicles [6], and the other two were healthy follicles of either an aligned/rounded or loopy/columnar phenotype [3]. The initial analyses across the three groups by one-way ANOVA did not indicate any gene differences with a minimum two fold change and False Discovery Rate (FDR), *P*<0.05, between the two groups of healthy follicles. Therefore these were treated as one group for further analyses (n = 10).

To exclude the possibility of contamination of the theca interna with granulosa cells, the array intensities of a granulosa cell marker, FSH receptor, was examined. All healthy thecal samples had signal intensities less than 3.0, compared with a mean ± SEM of 8.6±0.05 for granulosa cells (n = 10) from small healthy follicles of a similar size in an analogous study [19]. Therefore, considering that the signal data were log2 transformed the level of contamination by granulosa cells was estimated to be less than 2%.

**Figure 1:**
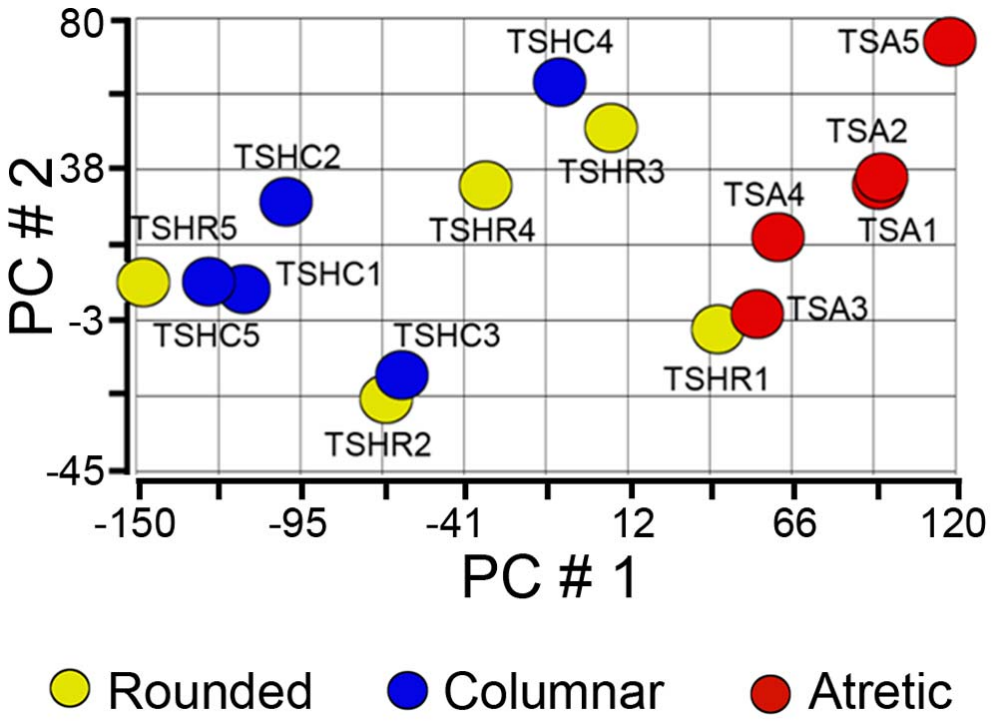
Unsupervised PCA of arrays from theca interna of small healthy and atretic follicles. The healthy follicles were separated into rounded (n = 5) and columnar (The graph is a scatter plot of the values for the first (X-axis) and second (Y-axis) principal components based on the Pearson correlation matrix of the total normalized array intensity data. The numbering of each sample enables the samples in this figure to be identified in Fig. S1. Abbreviations are: thecal sample healthy rounded (TSHR), thecal sample healthy columnar (TSHC) and thecal sample atretic (TSA).

PCA mapped the overall differences in gene expression between the individual arrays as shown in Fig. 1. Two array clusters were formed on the basis of follicle phenotype indicating that significant changes occur at the transcriptional level between atretic and healthy theca interna. Hierarchical clustering on the basis of gene expression between the arrays revealed a similar pattern, with partitioning related to follicle health (Fig. S1) though perhaps not as clearly defined as for the granulosa cells as shown by ourselves in a previous study [19]. This may be explained by the fact that the granulosa layer is a relatively homogeneous cell layer compared with the theca interna which contains steroidogenic cells, fibroblastic cells, immune cells and vasculature.

A total of 543 probe sets (out of 15,530 detected), was determined to be differentially expressed between the atretic and healthy theca interna (≥2-fold change, FDR *P*<0.05) by one-way ANOVA analyses in Partek (Table 2, further details in Table S1). This data set was considerably smaller than the equivalent group generated for granulosa cells (n = 5439) [19]. This suggests that the theca interna does not change its gene expression profile nearly to the same extent as granulosa cells during the initial stages of the atresia. This stability of the thecal transcriptome was also demonstrated recently by Christenson et al [7] who found fewer genes were changed in expression between the theca interna than were changed in the granulosa cells of larger follicles in response to LH. The n = 543 data set, consisting of 206 up regulated and 179 down regulated genes (Table 2), was uploaded for pathway and network analyses into Ingenuity Pathway Analysis (IPA) and into GOEAST software [21].

**Table 2:** Number of probe sets and genes differentially expressed in atretic compared with healthy follicles.

Fold-Change	Probe sets			Genes		
	Up Regulated	Down Regulated	Total	Up Regulated	Down Regulated	Total
>2	307	236	543	206	179	385
>3	52	112	164	37	90	127
>4	19	54	73	17	42	59

Determined by ANOVA with *P*<0.05 by the step-up Benjamini Hochberg FDR method for multiple corrections using Partek Genomics Suite Software.

### Functional and pathway analyses of differentially expressed genes

**Figure 2:**
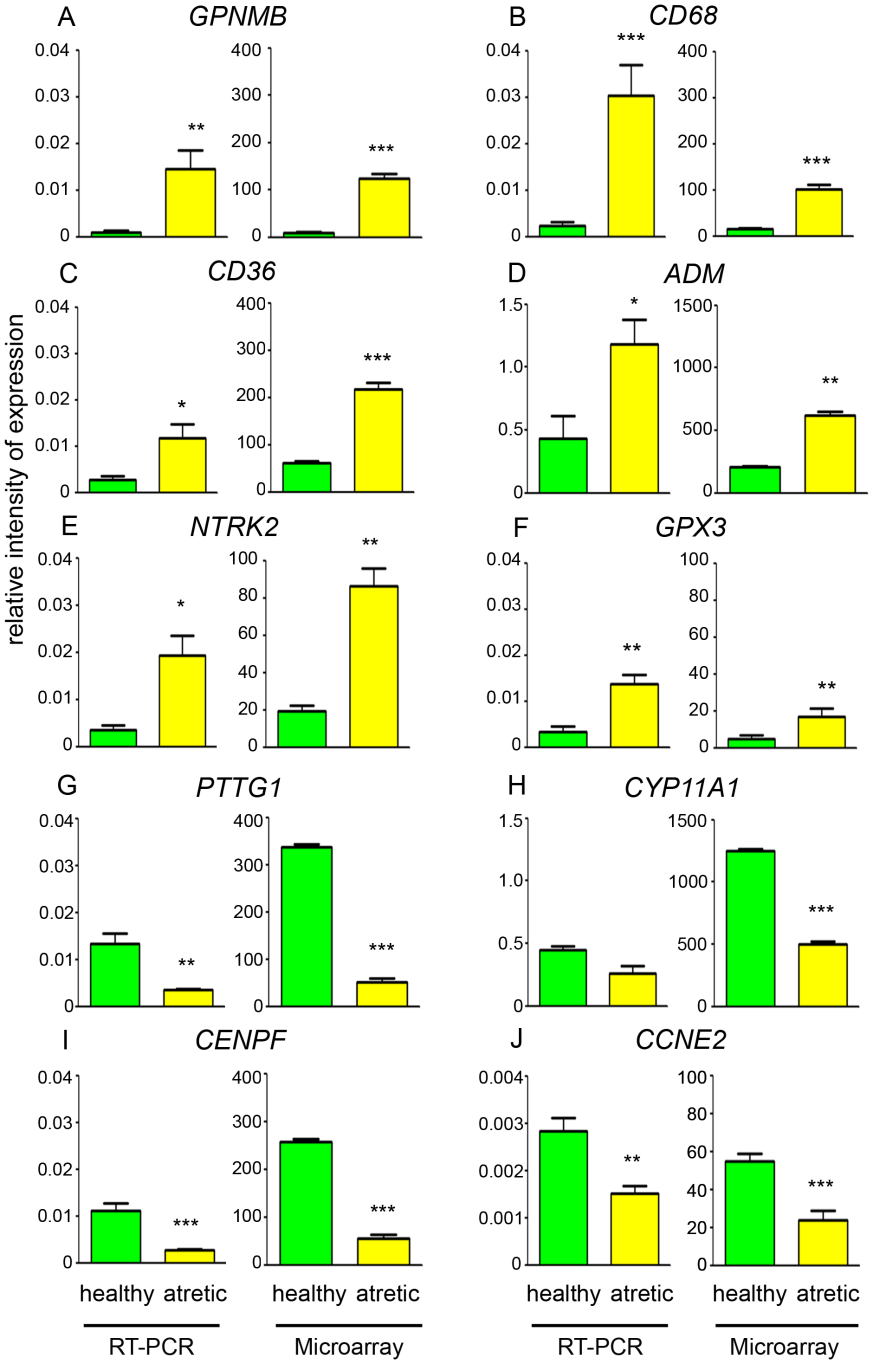
Validation of expression levels of genes in microarray by qRT-PCR. qRT-PCR gene expression values were determined from the mean of the ratio of the ΔCt for the target genes to cyclophilin A (*PPIA*) and glyceraldehyde phosphate dehydrogenase (GAPDH) and the data are mean ± SEM (n = 7 for each group). The microarray values are signal intensities (normalized but not log transformed, n = 10 healthy and n = 5 atretic samples). Significantly different results for qRT-PCR were determined by one-way ANOVA with Tukey's post-hoc test. The microarray signal intensity data were analyzed by ANOVA with corrections for multiple testing using the FDR. *P<0.05, **P<0 01 and ***P<0 001.

Three hundred and fifty three genes were mapped to the IPA knowledge base, which included 182 up- and 171 down-regulated genes in theca interna from atretic compared with healthy follicles. These genes were categorized by biological function and are listed in Tables 3 and 4. Ten genes which showed differential regulation and were associated with the key processes of inflammation, steroidogenesis and cell division were selected for validation (Fig. 2). The fold-change data from the arrays and the qRT-PCR experiments were highly correlated with each other (Pearson's correlation, *R* ^2^ = 0.929, *P*<0.001; Fig. S2), indicating that the arrays were correctly identifying differentially expressed genes.

**Table 3:** Genes up-regulated in atretic compared with healthy follicles.

Gene Symbol	Fold Change	Gene Symbol	Fold Change	Gene Symbol	Fold Change
**Cell Cycle and DNA Replication**					
ZSWIM7	2.2				
**Cell Death**					
NOL3	3.0	NDRG1	2.2		
RBM5	2.7				
**Cell Morphology**					
SAMSN1	2.9	NEBL	2.4	CORO1A	2.1
LCP1	2.5	MYH11	2.2		
**Cytokines, Hormones and Receptors**					
CD68	6.4	ANGPT2	2.8	GPR77	2.3
CD14	4.7	CD5L	2.7	IL1R1	2.3
NTRK2	4.5	IL6	2.6	CD86	2.2
CXCR4	3.9	NRP2	2.6	TLR7	2.2
CD36	3.5	PTGER4	2.5	LY9	2.2
TYROBP	3.5	MRC1	2.5	PLXNC1	2.1
MSR1	3.4	FCGR1A	2.5	GMFG	2.1
FOLR2	3.3	CD48	2.4	TGFBI	2.1
ADM	3.0	RYR2	2.4	CD84	2.1
CD53	2.9	LAPTM5	2.4	GPR34	2.1
CCR1	2.8	IL10RA	2.4	FCER1A	2.1
OSMR	2.8	TIMD4	2.3	HRH1	2.1
**Extracellular Matrix and Synthesis**					
SPOCK2	3.1	SMOC2	2.6	COL4A6	2.1
**Intercellular and Cell to Matrix Adhesion**					
LGALS3	5	CLDN5	2.5	ITGB5	2.2
CLDN11	4.6	SIGLEC1	2.2	ITGB2	2.2
VCAM1	2.5	FERMT3	2.2		
**Ion Transport**					
SLC11A1	2.2	PLN	2.2	SLC24A6	2
SLC31A2	2.2	SLC40A1	2.1		
**Protein Trafficking**					
CLU	2.1	ADCK3	2.0		
**Proteolysis or Inhibition**					
C1S	7.0	FGL2	2.7	PCOLCE2	2.1
CFI	5.1	UBE2L6	2.7	CTSZ	2.1
SERPING1	4.0	FBXO32	2.6	CNDP2	2.0
A2M	3.0	CSTB	2.6	LGMN	2.0
MMP19	3.0	CTSF	2.3		
CTSB	3.0	LTF	2.3		
**Transcription Regulation**					
CEBPD	4.2	CREBRF	2.5	PRDM1	2.2
NUPR1	3.6	RBPMS	2.3	KLF15	2.1
FOS	3.1	EGR1	2.3	GAS7	2.1
FOSL2	3.1	ZFP36	2.3	MXI1	2.1
JUN	2.6	CITED2	2.2	KANK1	2.0
BCL6	2.5	TGIF1	2.2		
**Transport**					
APOD	6.8	SNX31	2.5	CYBB	2.2
STARD10	2.7	ABCG1	2.5	KLHL24	2.1
SLC7A7	2.7	DYNLRB2	2.3	GABARAPL1	2.0
APOE	2.6	RTP4	2.3	ABCC8	2.0
**Other Enzymes**					
CH25H	4.1	GIMAP7	2.5	AMY2A	2.2
ATP2C2	3.6	STEAP1	2.4	PDK4	2.2
GPX3	3.4	HSD17B11	2.4	PTGDS	2.2
ACP5	3.2	ADCY8	2.4	GIMAP1-GIMAP5	2.2
CP	3.2	HMOX1	2.4	RNASE1	2.1
VNN1	2.9	MAN1A1	2.4	IQCA1	2.1
CHI3L2	2.8	ENPP2	2.3	GAMT	2.1
PLA1A	2.8	ALG13	2.3	PARP12	2.0
ASPA	2.6	NPL	2.2	RNASE6	2.0
MAOB	2.6	RENBP	2.2		
ATP9A	2.5	AKR1C3	2.2		
**Other Signalling**					
PKIB	4.5	RASGEF1B	2.7	ARHGEF6	2.2
APCDD1	3.1	ARHGEF11	2.4	SHISA2	2.2
RGS1	3.0	DUSP26	2.3	RRAD	2.2
MERTK	2.8	GEM	2.2		
SLAMF7	2.7	AIF1	2.2		
**Other**					
GPNMB	14.5	C1QB	2.7	WIPF3	2.2
SAA2	5.9	C21orf7	2.7	C19orf76	2.2
C7	4.4	MPEG1	2.6	TCP11L2	2.2
C10orf10	4.1	S100A13	2.5	MOB3B	2.2
SCUBE2	3.2	C1QA	2.5	WDFY4	2.2
PID1	3.0	FABP5	2.5	FAM210B	2.1
YPEL3	2.8	MXRA8	2.5	C1QTNF7	2.1
ISM1	2.8	TMEM156	2.3	C7orf41	2.1
C1QC	2.8	TMEM150C	2.3	FAM84A	2.1
MT1H	2.7	FGL1	2.3	FAM20A	2.0
**Non-IPA annotated genes**					
LOC504773	4.9	C13H20ORF12	2.5	LOC509513 /// TRB@	2.2
C1R	4.2	MRC1L1	2.4	SULT1A1	2.2
LOC783399	3.3	EPHX2 /// LOC785508	2.4	LOC100139766 /// LOC507	2.2
LOC784007	3.0	DCLK1	2.4	NKG7	2.2
VSIG4	2.9	LOC507141	2.3	PDPN	2.1
C4A	2.7	LOC618591	2.2	LOC513508	2.1
RGS2	2.7	N4BP2L1	2.2	LOC513587	2.1
LOC535166	2.6	CTSW	2.2	SLAMF9	2.1

≥2 fold-change with *P*<0.05 by Benjamini-Hochberg post-hoc test for multiple corrections following one-way ANOVA and categorized by function. Assignation of genes to categories was determined manually by the authors based on available information from NCBI databases and literature. Genes are listed in descending order of fold change within each category.

**Table 4:** Genes down-regulated in atretic compared with healthy follicles.

Gene Symbol	Fold Change	Gene Symbol	Fold Change	Gene Symbol	Fold Change
**Cell cycle and DNA replication**					
FAM64A	7.3	CKS2	4.0	CENPP	2.7
UHRF1	7.0	NCAPG	4.0	BRCA1	2.7
CCNB1	7.0	TPX2	3.9	ORC1	2.6
PTTG1	6.5	CDCA7	3.9	SMC4	2.6
CDCA8	6.2	CCDC99	3.8	PCNA	2.6
CENPN	5.8	E2F8	3.8	POLE2	2.6
CDCA2	5.7	SGOL1	3.8	CHTF18	2.5
RRM2	5.7	OIP5	3.8	SMC2	2.5
HJURP	5.6	KIF4A	3.8	CKS1B	2.5
RAD51AP1	5.5	FAM83D	3.7	MCM2	2.5
CDK1	5.5	KNTC1	3.6	UBE2S	2.4
ASPM	5.4	CCNF	3.6	ATAD5	2.4
TOP2A	5.4	ECT2	3.5	CDT1	2.4
CDCA5	5.4	KIF22	3.5	MCM5	2.4
AURKB	5.3	AURKA	3.5	CHAF1B	2.4
CDCA3	5.3	MAD2L1	3.4	HMGB2	2.3
ASF1B	5.2	MCM4	3.4	PLK1	2.3
ESPL1	5.2	CHAF1A	3.3	RPA3	2.3
BUB1	5.2	KIFC1	3.3	CCNE2	2.3
CDC20	5.2	SKA3	3.3	BORA	2.3
CCNB2	5.1	ERCC6L	3.2	PSRC1	2.3
KIF20A	5.1	CDC6	3.2	CENPO	2.2
NUSAP1	5.1	STIL	3.2	NDE1	2.2
KIF2C	5.0	RACGAP1	3.2	GINS2	2.2
CENPE	5.0	NCAPG2	3.2	GINS3	2.2
CASC5	5.0	FANCI	3.2	MCM6	2.2
SPC24	4.9	DSN1	3.2	STRA13	2.1
SPAG5	4.8	CKAP2	3.2	MYBL2	2.1
PRC1	4.7	MCM3	3.1	CDC25C	2.1
CCNA2	4.7	ZWINT	3.1	RCC1	2.1
CENPF	4.7	KIF23	3.0	NCAPD3	2.0
NCAPH	4.6	FEN1	2.9	H2AFZ	2.0
MELK	4.5	H2AFX	2.8	RRM1	2.0
SKA1	4.4	CHEK1	2.8	RPA2	2.0
BUB1B	4.2	VRK1	2.7	LIG1	2.0
NDC80	4.2	RMI2	2.7	NSL1	2.0
**Cell Death**					
BIRC5	5.7				
**Cell Morphology**					
CKAP2L	3.8	ANLN	2.9		
LMNB1	3.7	NRM	2.2		
**Cytokines, Hormones and Receptors**					
HMMR	3.6	CCL25	2.9	FGFR2	2.3
APLNR	3.2	VEGFA	2.7		
**Intercellular and Cell to Matrix Adhesion**					
TROAP	2.8	PCDH7	2.1		
**Ion Transport**					
KCNMA1	3.3				
**Protein Trafficking**					
KPNA2	3.7				
**RNA Processing**					
DDX39A	2.6	MAGOHB	2.1		
SRSF1	2.3	LSM4	2.0		
**Transcription Regulation**					
TRIP13	3.8	ARHGEF39	3.1	EZH2	2.1
TCF19	3.4	MXD3	3.0		
**Transport**					
SLC16A1	2.3	SLCO2A1	2.2	AQP11	2.2
**Other Enzymes**					
KIAA0101	7.0	CYP11A1	2.5	LIPG	2.2
UBE2C	6.0	TYMS	2.4	CYB5R3	2.2
TK1	3.2	DCK	2.3	PSAT1	2.1
DTYMK	3.1	ACOT7	2.3	PPA1	2.1
HPGD	3.0	ASS1	2.2	MTHFD1	2.0
PHGDH	2.7	ALPL	2.2	BDH1	2.0
DUT	2.6	CRYM	2.2		
**Other Signalling**					
SHCBP1	5.8	IQGAP3	2.7		
**Other**					
OIT3	3.9	C11orf82	2.3	RDM1	2.1
TFF2	2.9	S100A2	2.3	CXorf69	2.1
TMEM88	2.4	PLIN5	2.2	CCDC115	2.0
C1orf112	2.4	MANF	2.2	MRPL15	2.0
HN1	2.3	TAGLN3	2.2	BCS1L	2.0
**Non-IPA annotated genes**					
CENP-A /// LOC782601 //	5.2	FBXO5	3.2	FOXM1	2.1
CENP-A /// CENP-A /// L	3.9	TUBA1A /// TUBA1B	2.3		
TACC3	3.7	LOC100138846 /// LOC784	2.1		

≥2 fold-change with *P*<0.05 by Benjamini-Hochberg post-hoc test for multiple corrections following one-way ANOVA and categorized by function. Assignation of genes to categories was determined manually by the authors based on available information from NCBI databases and literature. Genes are listed in descending order of fold change within each category.

The most highly up-regulated gene was *GPNMB* (14-fold, Table 3, Fig. 2A). This gene was one of several in the data set of differentially expressed genes which were associated with inflammatory response, such as the complement components *C7*, *C1S*, *C1R* and *CF1*; the macrophage marker *CD68*, adrenomedullin (*ADM*) and glutathione peroxidase 3 (*GPX3*). Differential expression of C*D68*, *ADM* and *GPX3* were also validated by qRT-PCR as shown in Figs. 2B, D and F. A large number of genes in the up-regulated data set appear to be either cytokines, hormones or receptors (n = 36, Table 3). This group also contains many genes which map to inflammatory pathways including: *IL-6* (interleukin-6), interleukin receptors *IL1R1* and *IL10RA*, prostaglandin receptor *PTGER4*, Toll-like receptor 7 (*TLR7*), and the high affinity receptors for the Fc fragment of IgG and IgE, *FCGR1A* and *FCER1A*, respectively. Other genes of interest from this group which may mediate extracellular ligand actions were *NTRK2* (Table 3 and Fig. 2E), and *NRP2*. Another significant group of up-regulated genes in atretic follicles is represented by the transcription regulators (n = 17, Table 3). These include a number of important genes such as *FOS*, *EGR1*, *CEBPD* and *FOSL2* which can participate in regulation of steroidogenesis.

Numerous genes categorized by function present in the down-regulated data set (Table 4) were connected with cell division and DNA replication (n = 108). These included cell division cycle-associated genes, e.g *CDCA8* and *CDCA2*; cyclins, e.g. *CCNB1* and *CCNB2*; mini chromosome maintenance complex components e.g *MCM3* and *MCM4*, and kinesins. Two cell cycle associated genes, *CENPF* and *CCNE2* (Figs. 2I, J and F) were further validated by qRT-PCR. *PTTG1* (Fig. 2G) which encodes the mammalian homolog of securin was also down-regulated. It has important roles in cell cycle progression and has been associated with oocyte competence in other bovine array studies [24], [25].

**Figure 3:**
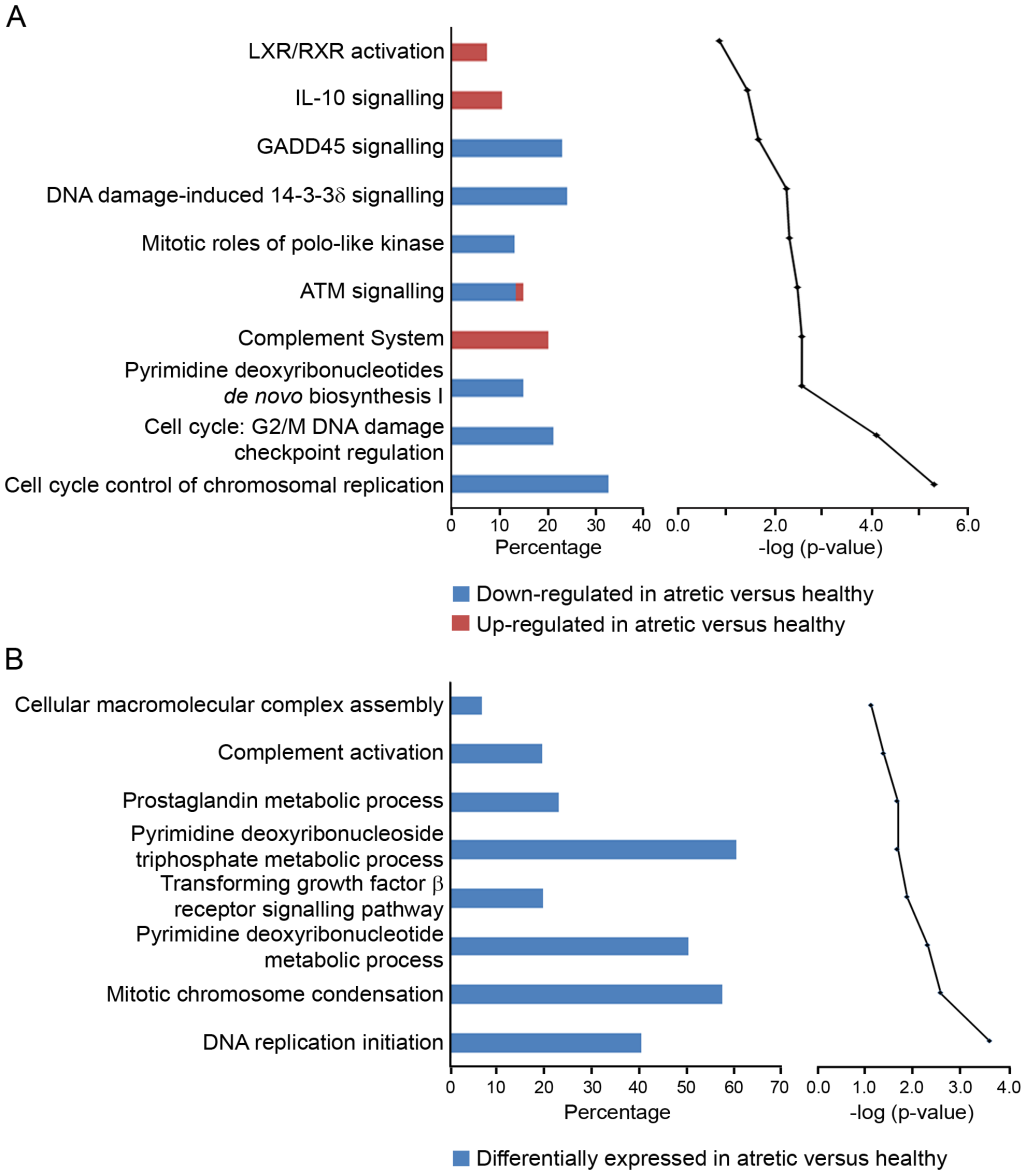
Top canonical pathways mapped in IPA (A) and GO terms (B) classified under biological process. Data set analysed were genes differentially regulated (2 fold with FDR *P*<0.05) between atretic and healthy samples. In (A) the bar chart on the left represents the percentage of genes from the data set that map to each canonical pathway, showing those which are up-regulated (in red) and down-regulated (in blue) in atretic compared with healthy follicles. The line chart on the right ranks these pathways, from the highest to lowest degree of association based on the value of Benjamini-Hochberg test for multiple corrections (bottom to top in graph on right). In (B) the bar chart on the left represents the proportion of genes which map to a GO term associated with a biological process. The line chart on the right ranks these pathways from the highest to lowest degree of association (bottom to top) using the Benjamini-Yuketeli test for multiple corrections.

IPA determined that six of the top ten canonical pathways which were associated with our data set were involved with cell division and DNA replication (Fig. 3A). These include, cell cycle control of chromosomal replication (*P* = 3.36×10^−6^), cell cycle: G2/M DNA damage checkpoint regulation (*P* = 4.96×10^−5^), pyrimidine deoxyribonucleotides *de novo* biosynthesis I (*P* = 1.63×10^−3^), mitotic roles of polo-like kinase (*P* = 2.90×10^−3^), DNA damage-induced 14-3-3δ signaling (*P* = 3.34×10^−3^) and GADD45 signaling (*P* = 1.24×10^−3^). The genes which mapped to these pathways were all down regulated, indicating inhibition of these processes. Additionally, the interleukin-10 (*P* = 2.08×10^−2^) and complement signaling pathways (*P* = 1.63×10^−3^) which are related to stress response and inflammation appeared to be activated. The GO enrichment analysis of our differentially expressed genes (Fig. 3B) also showed an emphasis on chromosomal and DNA metabolic processes e.g. mitotic chromosome condensation (*P* = 2.54×10^−3^) and pyrimidine deoxyribonucleotide metabolic process (*P* = 4.74×10^−3^), and some inflammation-related associations through complement (*P* = 3.94×10^−2^) and TGFβ pathways (*P* = 1.27×10^−2^).

**Figure 4:**
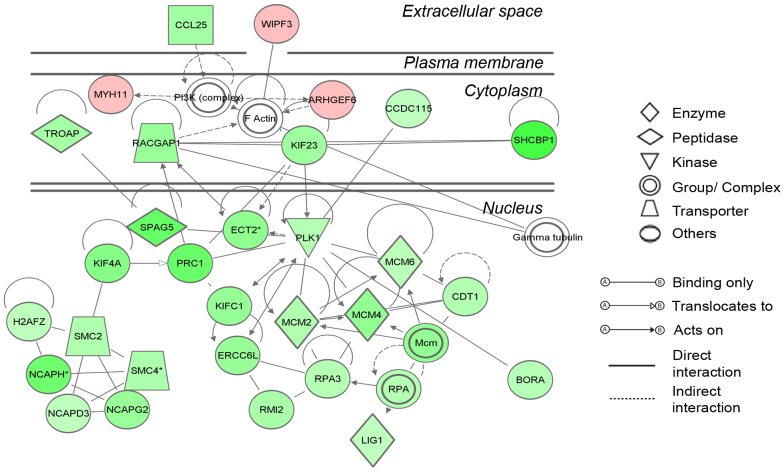
The most significant network determined in IPA. The network was generated in IPA using triangle connectivity based on focus genes (30 from our differentially regulated data set) and built up according to the number of interactions between a single prospective gene and others in the existing network, and the number of interactions the prospective gene has outside this network with other genes as determined by IPA [43]. Network score = 52, equivalent to −log *P* value of Fisher's exact *t*-Test. Interactions between molecules, and the degree and direction of regulation are indicated with up (red) or down regulation (green) and increasing color intensity with degree of fold change.

The most significant network generated by IPA from the differentially expressed genes is displayed in Fig. 4. This network showed that molecules mostly down regulated in atretic compared with healthy follicles mainly mapped to the nuclear compartment of the cells and were involved with chromosome organization as part of the cell cycle process. Genes which encode components of the condensin-2 complex such as *NCAPH*, *NCAPD3* and *NCAPG2* and the mini-chromosome complex which initiates DNA replication [26] such as *MCM2*, *MCM4* and *MCM6* were among these. This connection with cell division was further strengthened by the fact that the top canonical pathway in IPA associated with the differentially regulated data set was cell cycle control of chromosomal replication (Fig. S3).

### Predicted upstream regulators

Upstream regulators were predicted using a Fisher's exact *t*-test to determine the probability that genes from the dataset correspond with targets which are known to be activated or inhibited by those molecules based on current knowledge in the Ingenuity database. Analyses of predicted upstream regulators by IPA revealed that there was probable activation of the cyclin-dependent kinase inhibitor gene *CDKN2A* and the stress markers p53 and IKBKB (Table 5). Additionally, Vitamin D3, a steroidogenic regulator, the micro RNA let-7, and the oncogene *RB1*, were all also predicted to be activated in atretic thecal tissue. Upstream regulators which were predicted to be inhibited include cyclin D1, hepatocyte growth factor (HGF), estrogens and the transcription factors E2F1, EP400 and TBX2 (Table 6).

**Table 5:** The top six upstream regulators predicted by IPA to be activated in atretic versus healthy follicles.

Upstream Regulator	Activation z-Score†	*P* value of Overlap*	Target Molecules in Data Set
1-alpha, 25-dihydroxy vitamin D3	5.871	1.63E-31	*ANGPT2, ANLN, BRCA1, BUB1B, CCNA2, CCNB1, CD14, CDC20, CDC6, CDCA5, CDCA8, CDK1, CDT1, CEBPD, CHAF1A, CHAF1B, CHTF18, ECT2, EGR1, ESPL1, EZH2, FEN1, FOS, GEM, H2AFZ, HPGD, IL10RA, IL6, JUN, KIAA0101, KIF20A, KIF22, KIF23, KPNA2, LIG1, LTF, MAD2L1, MCM2, MCM3, MCM4, MCM5, MELK, MRC1, NRM, NUPR1, NUSAP1, PCNA, POLE2, PRC1, RACGAP1, RAD51AP1, RBPMS, RPA2, RRM2, SLC7A7, SMC2, SPAG5, TCF19, TK1, TPX2, VEGFA*
TP53	4.718	1.18E-30	*A2M, ADCK3, ANLN, APOE, ASF1B, ASPM, ASS1, AURKA, AURKB, BIRC5, BRCA1, BUB1, BUB1B, C11orf82, C1QC, CCDC99, CCNA2, CCNB1, CCNB2, CCNE2, CDC20, CDC25C, CDC6, CDK1, CDT1, CENPF, CHEK1, CKAP2, CKS1B, CLU, CP, CSTB, CTSB, CTSF, CYB5R3, DCK, DSN1, DUT, E2F8, EGR1, ENPP2, ESPL1, EZH2, FANCI, FEN1, FOS, GPX3, H2AFX, H2AFZ, HJURP, HMGB2, HMMR, HMOX1, IL6, ITGB5, JUN, KCNMA1, KIAA0101, KIF23, KNTC1, KPNA2, LGALS3, MAD2L1, MCM2, MCM3, MCM4, MCM5, MCM6, MT1H, MYBL2, NCAPD3, NCAPG, NCAPH, NDC80, NDRG1, NOL3, NUPR1, NUSAP1, PCDH7, PCNA, PLK1, POLE2, PRC1, PRDM1, PSRC1, PTGDS, RACGAP1, RAD51AP1, RRAD, RRM1, RRM2, S100A2, SERPING1, SLC16A1, SMC2, SMC4, TGFBI, TOP2A, TPX2, TYMS, UBE2C, UHRF1, VEGFA, VRK1*
let-7	5.452	1.35E-23	*AURKA, AURKB, BRCA1, BUB1, BUB1B, CCNA2, CCNB1, CCNE2, CCNF, CDC20, CDC6, CDCA2, CDCA3, CDCA5, CDCA8, CDK1, CDT1, CHEK1, CKS1B, E2F8, EZH2, MAD2L1, MCM2, MCM3, MCM4, MCM5, MCM6, ORC1, RRM1, RRM2*
RB1	3.133	1.23E-22	*ANGPT2, ASF1B, AURKB, BIRC5, BRCA1, CCNA2, CCNB1, CCNE2, CDC25C, CDC6, CDCA5, CDK1, CDT1, CHAF1A, CITED2, DCK, EGR1, EZH2, FEN1, FOS, HMGB2, IL6, LIG1, MCM2, MCM3, MCM4, MCM5, MCM6, MELK, MYBL2, ORC1, PCNA, PLK1, RAD51AP1, RRM1, RRM2, TCF19, TYMS, VEGFA, VRK1*
CDKN2A	4.513	2.20E-18	*ASF1B, AURKB, BIRC5, CCNA2, CDC25C, CDCA5, CDK1, CHAF1A, CITED2, DCK, EGR1, EZH2, FEN1, GAS7, HMGB2, IL6, ITGB5, JUN, KIFC1, MCM4, MCM5, MELK, MYBL2, PCNA, PLK1, RAD51AP1, RRM1, RRM2, TCF19, TGIF1, TK1, VEGFA, VRK1*
IKBKB	3.563	2.81E-17	*ACP5, AURKB, BRCA1, CCNA2, CCNE2, CCR1, CDC6, CEBPD, CH25H, CKS1B, CLU, CP, CTSB, CTSF, CTSZ, CXCR4, ECT2, EGR1, ENPP2, EZH2, FOS, H2AFX, HMOX1, IL6, ITGB5, KIF20A, KIF23, LMNB1,MT1H, PCDH7, PLK1, SAA2, VCAM1, VEGFA*

† The predicted activation state is inferred from the bias-corrected z-score. The bias-corrected z-score is computed based on the proportion of target genes present in the data set which are directionally regulated as expected according to known effects of the regulator on the target compiled from the literature.

*The *P* value of overlap measures the statistical significance of overlap using Fisher's exact *t*-test, between genes from the data set and those known to be acted upon by an upstream regulator.

**Table 6:** The top six upstream regulators predicted by IPA to be inhibited in atretic versus healthy follicles.

Upstream Regulator	Activation z-Score†	*P* Value of Overlap*	Target Molecules in Data Set
TBX2	−5.367	3.01E-26	*ANLN, ASF1B, AURKA, AURKB, BUB1, CCNA2, CDC6, CDCA3, CDCA5, CDK1, CDT1, CHAF1B, CHEK1, CKAP2, CKS1B, E2F8, EZH2, LIG1, MAD2L1, MCM2, MCM4, MCM5, MCM6, MXD3, NCAPG2, PLK1, PRC1, SGOL1, SMC2*
E2F1	−3.428	1.26E-24	*AMY2A, ANGPT2, AURKA, AURKB, BIRC5, BRCA1, CCNA2, CCNB1, CCNB2, CCNE2, CDC6, CDK1, CHEK1, CTSB, DUT, ECT2, EGR1, EZH2, FEN1, FGFR2, FOS, HMGB2, HN1, KIAA0101, MAD2L1, MCM2, MCM3, MCM4, MCM5, MCM6, MTHFD1, MYBL2, NDC80, ORC1, PCNA, PSAT1, RACGAP1, RPA2, RPA3, RRM1, RRM2, SMC4, SRSF1, TK1, TOP2A, TYMS, VCAM1, VEGFA, ZFP36*
EP400	−4.101	9.60E-24	*CCNA2, CCNF, CDC20, CDC6, CDCA3, CENPF, E2F8, H2AFZ, MCM3, MCM4, MYBL2, NCAPG2, PCNA, PLK1, PSRC1, RCC1, SGOL1, SKA1, UHRF1*
CCND1	−3.234	2.69E-16	*AURKA, BRCA1, C11orf82, C7, CASC5, CCNA2, CCNE2, CDC6, CDCA2, CDCA8, CENPF, CENPN, E2F8, ENPP2, FAM83D, HJURP, KIAA0101, KIF20A, KIF2C, KIF4A, KLHL24, MCM4, MELK, NCAPH, PCNA, RMI2, RRM2, TPX2, TRIP13, TYMS*
HGF	−3.040	2.07E-15	*A2M, ANGPT2, AURKA, AURKB, BIRC5, BUB1, BUB1B, CCNE2, CCNF, CDC20, CDC25C, CDC6, CDK1, CENPF, CKS1B, CXCR4, DTYMK, EGR1, FEN1, FOS, GEM, HMMR, HMOX1, IL6, KIF22, KIF2C, LGMN, MAD2L1, MCM2, MCM5, MELK, MERTK, NDC80, OSMR, PCNA, PLK1, PRC1, RCC1, SMC2, STIL, TPX2, TRIP13, UBE2C, VCAM1, VEGFA*
estrogen	−3.043	3.00E-12	*APOE, AURKA, BRCA1, CCNB2, CCNE2, CDC25C, CKS1B, CLU, CYBB, CYP11A1, EGR1, ESPL1, FEN1, FOS, IL6, JUN, LIG1, LTF, MCM3, MCM4, MCM5, PCNA, PTGDS, RRM2, SMC2, TOP2A, TYMS, UHRF1, VEGFA, ZFP36*

† The predicted activation state is inferred from the bias-corrected z-score. The bias-corrected z-score is computed based on the proportion of target genes present in the data set which are directionally regulated as expected according to known effects of the regulator on the target compiled from the literature.

*The *P* value of overlap measures the statistical significance of overlap using Fisher's exact *t*-test between genes from the data set and those known to be acted upon by an upstream regulator.

### Transcriptional processes of atresia in the theca interna

There is the possibility that changes in the level of transcripts between the healthy and atretic follicle types may have been be due to changes in the proportions of the different cell types within the theca interna, rather than a change in the transcriptome per se. However, in an *in vivo* study where follicle growth was monitored daily by ultrasound it was clear that the histological appearance of atresia with substantial death of the granulosa cells, developed rapidly and within 24 h [6]. Thus there would be little time for cellular composition of the theca interna to be substantially altered. Additionally the volume density of steroidogenic cells and endothelial cells even in advanced antral atresia is the same as healthy follicles [11]. Therefore it is unlikely that differential rates of cell division or death between different cell types in the theca interna would generate the changes in the transcriptome observed in the current experiment.

#### Inflammation

Several immune cell markers, including macrophage specific markers *CD68* and *CD14*, were highly expressed in the atretic follicles. *GPNMB* plays a central role in trafficking of phagocytic cell debris and is essential for tissue repair [27]. Macrophages and other antigen presenting cells are important for phagocytosis and signaling for recruitment of other immune cells in the ovary, e.g. lymphocytes in the atretic follicle as reviewed in [28]. More recently these have been shown to be necessary for proper follicular development [29]. The presence of up-regulated immunoregulatory molecules or those predicted to be active such as *TLR7*, *IKBKB*, and *IL6* and *TGFBI* further confirms this active inflammatory process in the theca interna during atresia. This picture of activated inflammatory pathways during atresia in small follicles reflects the situation seen at the molecular level in the granulosa cells [19].

*GPX3*, a glutathione peroxidase produced by the vascular endothelium, detoxifies oxygen radicals which can harm biomolecules leading to cell death [30]. An increase in *GPX3* expression in the small atretic follicles is consistent with higher levels of oxygen radicals present in the theca interna which may contribute to the induction of the atresia. EP400, predicted to be down-regulated here, has also been shown to be involved with metabolism of reactive oxygen species (ROS) [31], indicating that oxidative stress pathways may be affected.

#### Transcriptional regulation

There were several important transcription factors which were differentially regulated between small healthy and atretic follicle theca interna in our analyses. Some of these mentioned below were up regulated in atresia and are known to play a role in the control of steroidogenic pathways in thecal cells and other ovarian tissues. Interestingly c-Fos and EGR1 are key effectors of the Protein Kinase C pathway for modulation of androstenedione production via the action of *CYP17A1* [32]. *CEBPD* is a transcription factor which is involved with regulation of cell division and appears to be playing a similar role here [33]. *FOSL2* and *JUN* encode components of the AP-1 transcription complex which participates in the terminal differentiation of granulosa cells to luteal cells [34]. It is known that *RB1* controls *E2F1* transcription and that *RB1* plays a role in regulating follicular development in mice [35], it may also be acting in the theca here as predicted by the regulator analysis. These changes in transcription factor levels may mediate the inhibition of cell growth and metabolism seen in the theca interna as part of the process of atresia.

#### Angiogenesis

A few of the genes which were down regulated in atretic follicles have a positive role in angiogenesis such as *VEGF* and *FGFR2*, as reviewed in [36]. This could contribute to a decrease in vascular function within the theca interna which eventually accompanies follicle regression.

#### Cell cycle/DNA replication and cell death

It is obvious that the main effect of atresia in the theca interna of small follicles at the transcriptome level, was the considerable inhibition of the processes of cell division and DNA replication. This may be due to the predicted activation of p53, revealed by the upstream regulator analyses in Table 6. p53 either triggers apoptosis, or inhibits cell cycle progression depending on the degree and length of time of exposure to cell stress stimuli, as reviewed recently [37]. It would appear that the process of antral atresia in the theca interna is secondary to cell death in the membrana granulosa, which already shows morphological and molecular characteristics of cell death much earlier than in the theca interna [6], [11], [38]–[40]. It is probable that these observed changes in gene expression are due to a decrease in stimulating factors from the dying granulosa cells such as AMH or inhibin/activin(s) as reviewed by Knight et al [41]. In this form of early antral atresia, the theca interna is still capable of secreting androgens [10]. The changes seen here at the transcriptional level, suggest that as the follicle continues to regress these cells will either die by some non-apoptotic mechanism or lose their steroidogenic capacity and differentiate into a more fibroblastic phenotype.

## Conclusions

There were very few indicators of cell death observed in the theca interna of antral atretic follicles in our array analyses. This prompts the question as to whether significant cell death actually occurs early in the theca interna layer in antral atretic follicles. In the literature there is ample evidence of cell death in the theca interna occurring early in basal atretic follicles [10], [11] where clearly the theca interna behaves differently in atresia to the antral type. Our study suggests that antral atresia in the theca interna from small antral follicles is associated mainly with the arrest of cell cycle and DNA replication rather than up-regulation of apoptosis-associated genes as occurs in granulosa cell death [42]. Additionally up-regulation of inflammation and loss of angiogenic functions occurs.

## Supporting Information

Figure S1 **Unsupervised hierarchical clustering across all probe sets and arrays.** Probe sets (n = 24,182) and arrays (n = 15) were clustered using the Euclidian dissimilarity algorithm with the average linkage method in Partek Genomics Suite. The heatmap represents the distribution of normalized signal intensity, grouping by pattern similarity for both probe set and array. Abbreviations are explained in Fig. 1.
(TIF)

Figure S2 **Scatter plot of fold-changes in microarray intensity versus expression determined by qRT-PCR.** Values represent 10 selected genes as presented in Fig. 2. The two sets of data were highly correlated with each other (Pearson's correlation, *R^2^* = 0.93, *P*<0.001).
(PDF)

Figure S3 **The canonical pathway of cell cycle control of chromosomal replication in IPA.** Genes which were down regulated in small atretic follicles are in green and the degree of fold difference is commensurate with the color intensity. Benjamini-Hochberg FDR *P* value of gene association with pathway = 3.36×10^−6^.
(TIF)

Table S1 **Probe sets which were 2-fold or more up regulated in atretic with respect to healthy follicles.** Analyses were carried out by ANOVA in Partek with *P*<0.05 (n = 543) and assignations are presented in alphabetical order. Probe sets which did not have gene assignations are placed at the end of the list. The *P* value for multiple corrections was determined by the step-up FDR method.
(PDF)
