# Supplementary figures and images for: Transcriptome Profiling of the Theca Interna from Bovine Ovarian Follicles during Atresia
Source: PLoS One. 2014 Jun 23;9(6):e99706. doi: 10.1371/journal.pone.0099706 (PMC4067288; doi:10.1371/journal.pone.0099706)

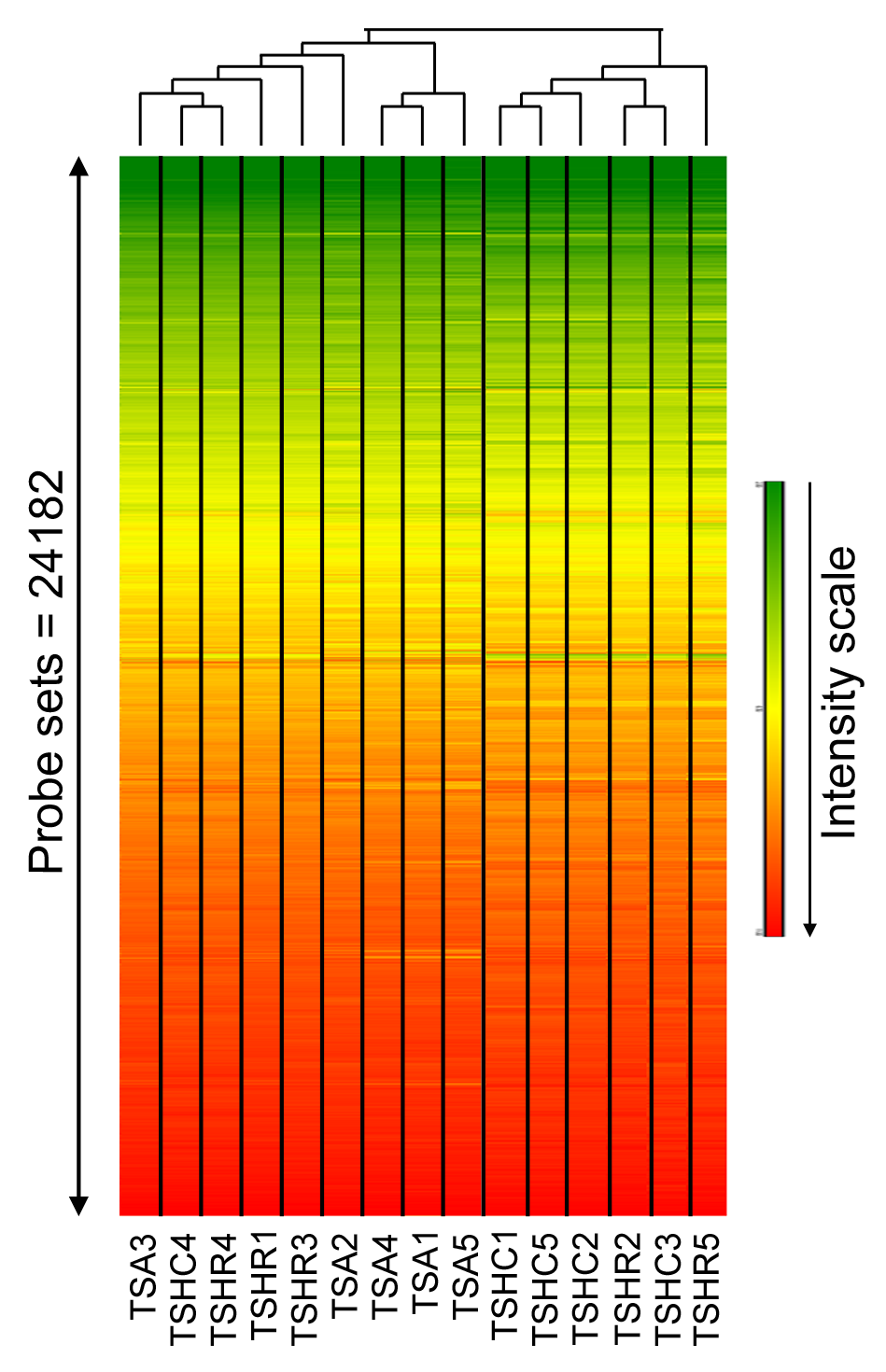

Supplement: Figure S1 — Unsupervised hierarchical clustering across all probe sets and arrays. Probe sets (n = 24,182) and arrays (n = 15) were clustered using the Euclidian dissimilarity algorithm with the average linkage method in Partek Genomics Suite. The heatmap represents the distribution of normalized signal intensity, grouping by pattern similarity for both probe set and array. Abbreviations are explained in Fig. 1. (TIF) [file pone.0099706.s001.tif]

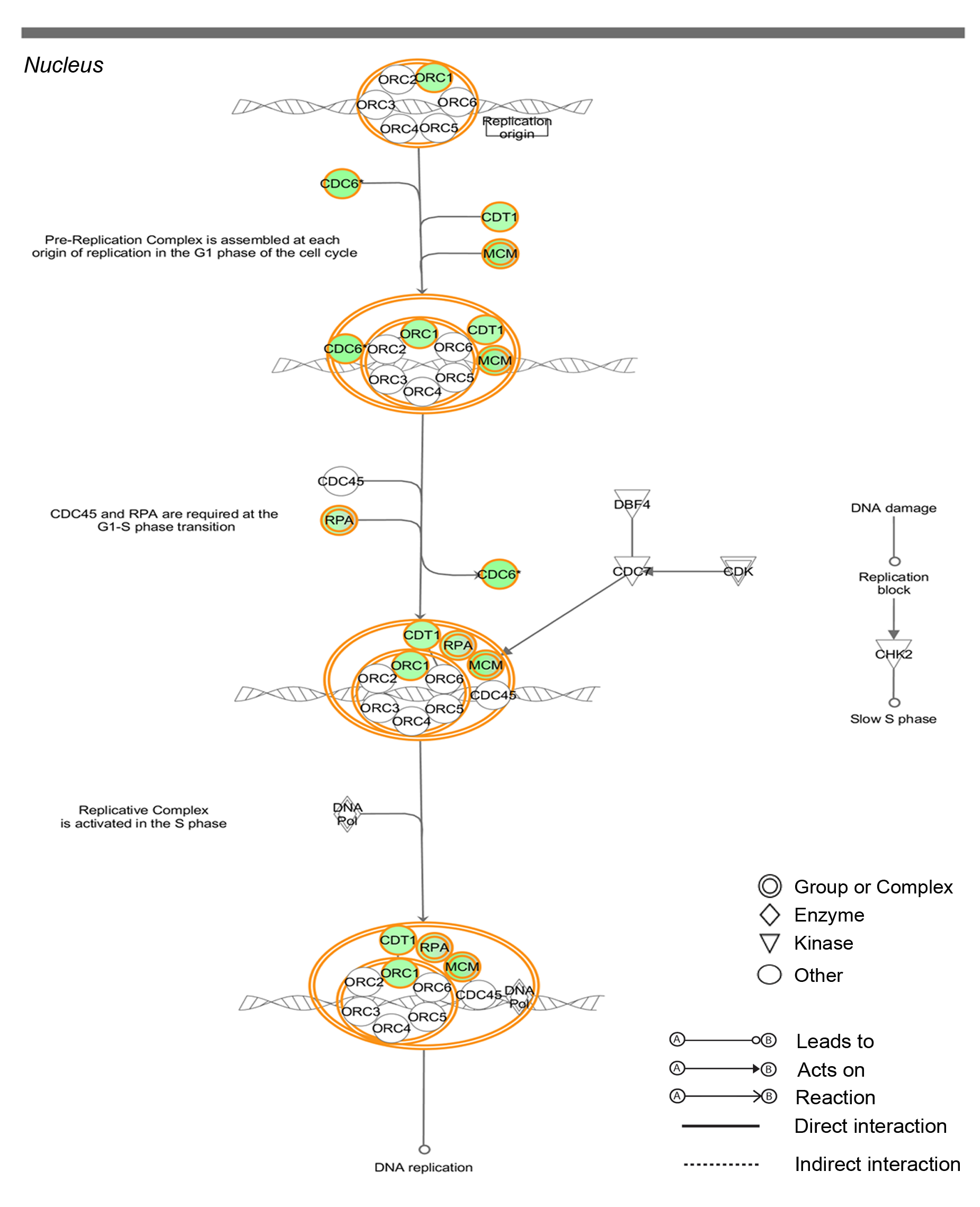

Supplement: Figure S3 — The canonical pathway of cell cycle control of chromosomal replication in IPA. Genes which were down regulated in small atretic follicles are in green and the degree of fold difference is commensurate with the color intensity. Benjamini-Hochberg FDR P value of gene association with pathway = 3.36×10^−6^. (TIF) [file pone.0099706.s003.tif]
