# Supplementary material for: Transcriptome Profiling of the Theca Interna from Bovine Ovarian Follicles during Atresia
Source: PLoS One. 2014 Jun 23;9(6):e99706. doi: 10.1371/journal.pone.0099706 (PMC4067288; doi:10.1371/journal.pone.0099706)

Scatter plot comparing fold-change values from microarray and qRT-PCR analysis (Pearsons correlation R^2 = 0.929, P < 0.001).

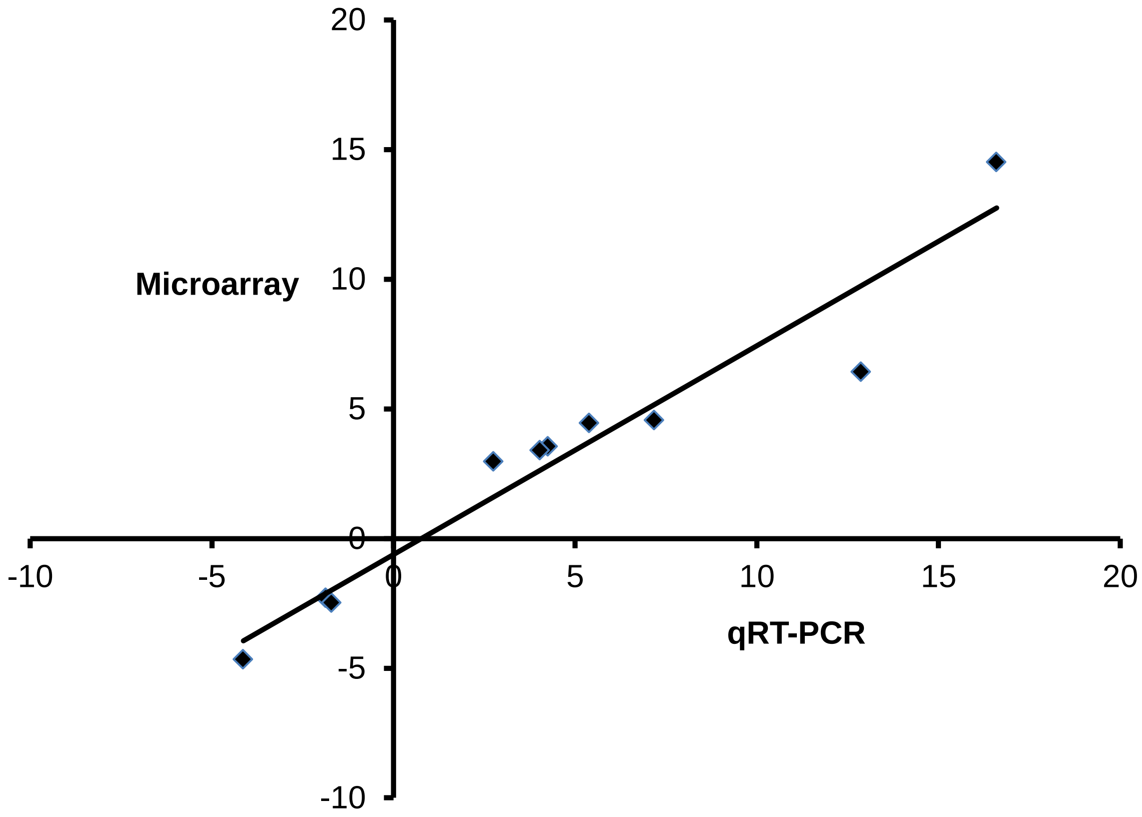

Supplement: Figure S2 — Scatter plot of fold-changes in microarray intensity versus expression determined by qRT-PCR. Values represent 10 selected genes as presented in Fig. 2. The two sets of data were highly correlated with each other (Pearson's correlation, R2 = 0.93, P<0.001). (PDF) [file pone.0099706.s002.pdf]
