# Supplementary material for: Transcriptome Profiling of the Theca Interna from Bovine Ovarian Follicles during Atresia
Source: PLoS One. 2014 Jun 23;9(6):e99706. doi: 10.1371/journal.pone.0099706 (PMC4067288; doi:10.1371/journal.pone.0099706)
Supplement: Table S1 — Probe sets which were 2-fold or more up regulated in atretic with respect to healthy follicles. Analyses were carried out by ANOVA in Partek with P<0.05 (n = 543) and assignations are presented in alphabetical order. Probe sets which did not have gene assignations are placed at the end of the list. The P value for multiple corrections was determined by the step-up FDR method. (PDF) [file pone.0099706.s004.pdf]

**Table S1.** Probe sets which are differentially regulated in atretic with respect to healthy follicles.

| Probe set ID | Gene Symbol | Gene Title | RefSeq Transcript ID | Fold Change | *P* Value |
|---|---|---|---|---|---|
| Bt.13326.1.S1_at | *A2M* | alpha-2-macroglobulin | NM_001109795 | 3.018 | 9.29E-03 |
| Bt.19199.1.A1_at | *ABCC8* | ATP-binding cassette, sub-family C (CFTR/MRP), member 8 | XM_584132 | 2.046 | 3.84E-02 |
| Bt.21471.1.S1_at | *ABCG1* | ATP-binding cassette, sub-family G (WHITE), member 1 | XM_587930 | 2.475 | 1.49E-02 |
| Bt.13293.1.S1_at | *ACOT7* | acyl-CoA thioesterase 7 | NM_001075682 | -2.308 | 1.57E-02 |
| Bt.19884.2.S1_at | *ACP5* | acid phosphatase 5, tartrate resistant | XM_595165 | 3.212 | 5.24E-03 |
| Bt.5215.1.S1_at | *ACP5* | acid phosphatase 5, tartrate resistant | XM_595165 | 2.385 | 6.10E-03 |
| Bt.2657.1.S1_a_at | *ADCY8* | adenylate cyclase 8 (brain) | XM_001249965 /// XM_614987 | 2.387 | 1.95E-02 |
| Bt.6202.1.S1_at | *ADM* | adrenomedullin | NM_173888 | 2.961 | 2.12E-02 |
| Bt.23658.2.S1_at | *AIF1* | allograft inflammatory factor 1 | NM_173985 | 2.244 | 6.36E-03 |

| | | | | | |
|---|---|---|---|---|---|
| Bt.27319.1.A1_at | *ALG13* | asparagine-linked glycosylation 13 homolog (S. cerevisiae) | NM_001080275 | 2.263 | 3.20E-02 |
| Bt.5200.1.S1_at | *ALPL* | alkaline phosphatase, liver/bone/kidney | NM_176858 | -2.211 | 2.30E-02 |
| Bt.4507.1.S1_at | *AMY2A* | amylase, alpha 2A (pancreatic) | NM_001035016 | 2.208 | 2.47E-02 |
| Bt.16232.1.S1_at | *ANGPT2* | angiopoietin 2 | NM_001098855 | 2.812 | 1.82E-02 |
| Bt.20272.1.S1_at | *ANLN* | Anillin, actin binding protein | XM_596461 | -2.663 | 9.03E-03 |
| Bt.4863.1.S1_at | *ANLN* | anillin, actin binding protein | XM_596461 | -2.889 | 1.84E-02 |
| Bt.2112.1.S1_at | *APCDD1* | Adenomatosis polyposis coli down-regulated 1 | XM_585921 | 3.127 | 1.04E-02 |
| Bt.24830.1.A1_at | *APLNR* | apelin receptor | NM_001102524 | -2.084 | 4.53E-03 |
| Bt.3686.1.S1_at | *APLNR* | apelin receptor | NM_001102524 | -3.199 | 4.91E-03 |
| Bt.11936.1.S1_a_at | *APOD* | Apolipoprotein D | NM_001076301 | 6.763 | 5.16E-03 |
| Bt.16622.1.A1_at | *APOD* | Apolipoprotein D | NM_001076301 | 3.734 | 6.18E-03 |
| Bt.8491.1.S1_at | *APOD* | apolipoprotein D | NM_001076301 | 2.808 | 6.94E-03 |

| | | | | | |
|---|---|---|---|---|---|
| Bt.4733.1.S1_at | *APOE* | apolipoprotein E | NM_173991 | 2.553 | 5.84E-03 |
| Bt.21513.2.S1_at | *AQP11* | aquaporin 11 | NM_001110069 | -2.196 | 1.36E-02 |
| Bt.10007.1.A1_at | *ARHGEF11* | Rho guanine nucleotide exchange factor (GEF) 11 | XM_588515 | 2.437 | 9.70E-03 |
| Bt.26011.1.A1_at | *ARHGEF6* | Rac/Cdc42 guanine nucleotide exchange factor (GEF) 6 | XM_865157 | 2.191 | 1.40E-02 |
| Bt.666.1.S1_at | *ASF1B* | ASF1 anti-silencing function 1 homolog B (S. cerevisiae) | NM_001075453 | -2.950 | 7.64E-03 |
| Bt.21523.1.S1_at | *ASF1B* | ASF1 anti-silencing function 1 homolog B (S. cerevisiae) | NM_001075453 | -5.224 | 5.49E-03 |
| Bt.6009.1.S1_at | *ASPA* | aspartoacylase (Canavan disease) | NM_001046033 | 2.631 | 5.16E-03 |
| Bt.16721.1.A1_at | *ASPM* | asp (abnormal spindle) homolog, microcephaly associated (Drosophila) | XM_614763 | -5.480 | 4.91E-03 |
| Bt.26895.1.S1_at | *ASS1* | argininosuccinate synthase 1 | NM_173892 | -2.228 | 4.55E-03 |
| Bt.21028.1.S1_at | *ATAD5* | ATPase family, AAA domain containing 5 | XM_590682 | -2.173 | 3.57E-02 |
| Bt.11428.1.A1_at | *ATAD5* | ATPase family, AAA domain containing 5 | XM_590682 | -2.419 | 2.73E-02 |
| Bt.16725.2.A1_at | *ATP2C2* | ATPase, Ca++ transporting, type 2C, member 2 | XM_587457 | 3.561 | 5.02E-03 |

| | | | | | |
|---|---|---|---|---|---|
| Bt.21433.2.S1_at | *AURKA* | aurora kinase A | NM_001038028 | -3.167 | 7.03E-03 |
| Bt.9510.2.A1_a_at | *AURKA* | aurora kinase A | NM_001038028 | -3.459 | 9.47E-03 |
| Bt.20521.3.A1_at | *AURKB* | aurora kinase B | NM_183084 | -3.047 | 4.91E-03 |
| Bt.16047.1.S1_at | *AURKB* | aurora kinase B | NM_183084 | -5.310 | 4.83E-03 |
| Bt.28185.1.S1_at | *BCL6* | B-cell CLL/lymphoma 6 | XM_584289 /// XM_865691 | 2.502 | 1.25E-02 |
| Bt.4198.1.S1_at | *BCS1L* | BCS1-like (yeast) | NM_001015671 | -2.002 | 1.30E-02 |
| Bt.10503.1.S1_at | *BDH1* | 3-hydroxybutyrate dehydrogenase, type 1 | NM_001034600 | -2.027 | 3.26E-02 |
| Bt.17939.1.S1_at | *BIRC5* | baculoviral IAP repeat-containing 5 | NM_001001855 | -5.720 | 5.24E-03 |
| Bt.21540.1.S1_at | *BORA* | aurora borealis | XM_591968 | -2.283 | 4.58E-03 |
| Bt.21294.1.A1_at | *BRB* | brain ribonuclease | NM_173891 | 2.093 | 4.14E-02 |
| Bt.12891.1.S1_at | *BRCA1* | Breast cancer 1, early onset | NM_178573 | -2.650 | 1.46E-02 |
| Bt.27757.1.S1_at | *BUB1* | budding uninhibited by benzimidazoles 1 homolog (yeast) | NM_001102011 | -2.002 | 1.45E-02 |

| | | | | | |
|---|---|---|---|---|---|
| Bt.17992.1.S1_at | *BUB1* | budding uninhibited by benzimidazoles 1 homolog (yeast) | NM_001102011 | -5.208 | 5.35E-03 |
| Bt.16875.1.A1_at | *BUB1B* | budding uninhibited by benzimidazoles 1 homolog beta (yeast) | NM_001145173 /// XM_617181 | -4.217 | 5.16E-03 |
| Bt.13204.1.S1_at | *C13H20ORF108* | chromosome 20 open reading frame 108 ortholog | NM_001080278 | 2.109 | 5.63E-03 |
| Bt.27043.1.A1_at | *C13H20ORF12* | chromosome 20 open reading frame 12 ortholog | NM_001076838 | 2.505 | 9.28E-03 |
| Bt.21886.1.S1_at | *C1H21ORF7* | chromosome 21 open reading frame 7 ortholog | XM_613553 | 2.715 | 1.74E-02 |
| Bt.1190.1.A1_at | *C1QA* | complement component 1, q subcomponent, A chain | NM_001014945 | 2.525 | 6.91E-03 |
| Bt.24238.1.A1_at | *C1QB* | complement component 1, q subcomponent, B chain | NM_001046599 | 2.730 | 5.61E-03 |
| Bt.23532.1.S1_at | *C1QC* | complement component 1, q subcomponent, C chain | XM_587039 | 2.786 | 6.22E-03 |
| Bt.4872.1.S1_at | *C1QTNF7* | C1q and tumor necrosis factor related protein 7 | NM_001076201 | 2.075 | 2.45E-02 |
| Bt.20501.1.S1_at | *C1R* | complement component 1, r subcomponent | NM_001034407 | 4.172 | 5.49E-03 |
| Bt.24446.1.A1_at | *C1S* | complement component 1, s subcomponent | NM_001076550 | 7.038 | 5.45E-03 |
| Bt.9175.1.A1_at | *C28H10ORF10* | chromosome 10 open reading frame 10 ortholog | NM_001046515 | 4.122 | 6.99E-03 |

| | | | | | |
|---|---|---|---|---|---|
| Bt.13796.1.S1_at | *C4A* | complement component 4A | NM_001166485 /// XM_584742 | 2.736 | 7.30E-03 |
| Bt.23521.1.S1_at | *C7* | complement component 7 | NM_001045966 | 4.378 | 6.92E-03 |
| Bt.18895.1.A1_at | *C9orf100* | vav-like protein C9orf100 homolog | NM_001079800 | -3.122 | 1.32E-02 |
| Bt.7190.1.S1_at | *CABC1* | chaperone, ABC1 activity of bc1 complex homolog (S. pombe) | NM_001046419 | 2.031 | 1.08E-02 |
| Bt.3191.1.A1_at | *CASC5* | cancer susceptibility candidate 5 | XM_001790629 | -4.950 | 5.45E-03 |
| Bt.27207.1.A1_at | *CCDC115* | coiled-coil domain containing 115 | NM_001034444 | -2.024 | 7.74E-03 |
| Bt.719.1.A1_at | *CCDC99* | coiled-coil domain containing 99 | NM_001076854 | -3.834 | 9.91E-03 |
| Bt.2047.1.S1_at | *CCL25* | chemokine (C-C motif) ligand 25 | NM_001046569 | -2.892 | 1.95E-02 |
| Bt.4310.1.A1_at | *CCNA2 /// LOC786906* | cyclin A2 /// similar to Cyclin A2 | NM_001075123 /// XR_042775 | -4.669 | 5.99E-03 |
| Bt.20220.1.S1_at | *CCNB1* | cyclin B1 | NM_001045872 | -7.018 | 5.53E-03 |
| Bt.28541.1.S1_at | *CCNB2* | cyclin B2 | NM_174264 | -5.139 | 4.91E-03 |
| Bt.17994.1.S1_at | *CCNE2* | cyclin E2 | NM_001015665 | -2.296 | 8.91E-03 |

| | | | | | |
|---|---|---|---|---|---|
| Bt.16886.1.A1_a_at | *CCNF* | cyclin F | NM_001098870 | -3.611 | 5.24E-03 |
| Bt.4873.1.S1_at | *CCR1* | chemokine (C-C motif) receptor 1 | NM_001077839 | 2.834 | 4.55E-03 |
| Bt.25823.1.A1_at | *CD14* | CD14 molecule | NM_174008 | 4.686 | 5.24E-03 |
| Bt.24258.2.S1_at | *CD14* | CD14 molecule | NM_174008 | 2.393 | 6.36E-03 |
| Bt.24707.1.S1_at | *CD36* | CD36 molecule (thrombospondin receptor) | NM_174010 | 3.546 | 1.09E-02 |
| Bt.20827.1.A1_at | *CD48* | CD48 molecule | NM_001046002 | 2.418 | 1.89E-02 |
| Bt.8834.1.S1_at | *CD53* | CD53 molecule | NM_001034232 | 2.912 | 1.21E-02 |
| Bt.16094.1.S1_at | *CD5L* | CD5 molecule-like | NM_001102119 | 2.708 | 3.05E-02 |
| Bt.18639.1.A1_at | *CD68* | CD68 molecule | NM_001045902 | 6.423 | 5.06E-03 |
| Bt.26844.1.A1_at | *CD84* | CD84 molecule | XM_588136 | 2.116 | 2.22E-02 |
| Bt.13798.1.S1_at | *CD86* | CD86 molecule | NM_001038017 | 2.236 | 4.55E-03 |
| Bt.12685.1.S1_at | *CDC20* | cell division cycle 20 homolog (S. cerevisiae) | NM_001082436 | -5.193 | 4.91E-03 |

| | | | | | |
|---|---|---|---|---|---|
| Bt.18500.1.A1_at | *CDC25C* | cell division cycle 25 homolog C (S. pombe) | NM_001097996 | -2.098 | 9.71E-03 |
| Bt.7043.2.S1_a_at | *CDC6* | cell division cycle 6 homolog (S. cerevisiae) | XM_593299 | -2.222 | 2.10E-02 |
| Bt.28797.2.S1_at | *CDC6* | cell division cycle 6 homolog (S. cerevisiae) | XM_593299 | -3.220 | 2.31E-02 |
| Bt.546.1.S1_at | *CDCA2* | cell division cycle associated 2 | NM_001046194 | -5.729 | 4.91E-03 |
| Bt.28379.1.S1_at | *CDCA3* | cell division cycle associated 3 | NM_001035449 | -5.264 | 4.83E-03 |
| Bt.1181.1.S1_at | *CDCA5* | cell division cycle associated 5 | NM_001076270 | -5.371 | 5.86E-03 |
| Bt.24288.2.S1_at | *CDCA7* | cell division cycle associated 7 | NM_001037488 | -3.853 | 1.67E-02 |
| Bt.26844.2.S1_at | *CDCA8* | cell division cycle associated 8 | NM_001083652 | -2.701 | 1.05E-02 |
| Bt.25924.1.A1_at | *CDCA8* | cell division cycle associated 8 | NM_001083652 | -6.156 | 6.57E-03 |
| Bt.23453.1.S1_at | *CDK1* | cyclin-dependent kinase 1 | NM_174016 | -5.495 | 6.17E-03 |
| Bt.29477.1.A1_at | *CDT1* | chromatin licensing and DNA replication factor 1 | XM_597224 | -2.413 | 1.20E-02 |
| Bt.12565.1.S1_at | *CEBPD* | CCAAT/enhancer binding protein (C/EBP), delta | NM_174267 | 4.176 | 9.49E-03 |

| | | | | | |
|---|---|---|---|---|---|
| Bt.18639.2.S1_at | *CEBPD* | CCAAT/enhancer binding protein (C/EBP), delta | NM_174267 | 3.270 | 1.06E-02 |
| Bt.27849.2.S1_a_at | *CENP-A /// CENP-A /// LOC618307 /// LOC782601 /// LOC782634 /// LOC782883* | centromere protein-A /// CENP-A protein /// similar to Histone H3-like centromer | XM_001250060 /// XM_001250108 /// XM_001250161 /// XM_001250208 /// XM_001788955 | -3.898 | 4.91E-03 |
| Bt.29005.1.A1_at | *CENP-A /// LOC782601 /// LOC782883* | CENP-A protein /// similar to Histone H3-like centromeric protein A (Centromere | XM_001250060 /// XM_001250108 /// XM_001788955 /// XR_027462 | -5.217 | 4.83E-03 |
| Bt.1074.1.A1_a_at | *CENPF* | centromere protein F, 350/400ka (mitosin) | XM_612376 | -4.666 | 4.91E-03 |
| Bt.27798.1.A1_at | *CENPN* | centromere protein N | NM_001038099 | -3.937 | 7.03E-03 |
| Bt.26157.1.A1_at | *CENPN* | centromere protein N | NM_001038099 | -4.915 | 5.45E-03 |
| Bt.13670.1.S2_at | *CENPN* | centromere protein N | NM_001038099 | -5.759 | 6.74E-03 |
| Bt.24859.2.S1_at | *CENPO* | centromere protein O | NM_001077003 | -2.198 | 1.78E-02 |
| Bt.24266.1.A1_at | *CENPP* | centromere protein P | NM_001105615 | -2.710 | 1.34E-02 |

| | | | | | |
|---|---|---|---|---|---|
| Bt.17926.1.A1_at | *CFI* | complement factor I | NM_001038096 | 5.113 | 4.91E-03 |
| Bt.18116.3.A1_s_at | *CH25H* | cholesterol 25-hydroxylase | NM_001075243 | 4.124 | 1.69E-02 |
| Bt.12465.1.A1_at | *CHAF1A* | chromatin assembly factor 1, subunit A (p150) | NM_001101842 | -3.311 | 1.08E-02 |
| Bt.1052.1.A1_at | *CHAF1B* | chromatin assembly factor 1, subunit B (p60) | NM_001098014 | -2.364 | 2.26E-02 |
| Bt.13546.1.A1_at | *CHEK1* | CHK1 checkpoint homolog (S. pombe) | NM_001098023 | -2.752 | 2.63E-02 |
| Bt.7588.1.A1_at | *CHI3L2* | similar to chitinase 3-like 2 | XR_042527 | 2.826 | 1.30E-02 |
| Bt.24354.1.S1_at | *CHTF18* | CTF18, chromosome transmission fidelity factor 18 homolog (S. cerevisiae) | XM_595170 | -2.505 | 2.22E-02 |
| Bt.4615.1.S1_at | *CITED2* | Cbp/p300-interacting transactivator, with Glu/Asp-rich carboxy-terminal domain, | NM_001075819 | 2.209 | 2.18E-02 |
| Bt.26029.1.A1_at | *CKAP2* | cytoskeleton associated protein 2 | NM_001098032 | -3.153 | 5.86E-03 |
| Bt.22389.1.S1_at | *CKAP2L* | cytoskeleton associated protein 2-like | NM_001098901 /// XM_584116 | -3.843 | 5.24E-03 |
| Bt.19204.1.S1_at | *CKS1B* | CDC28 protein kinase regulatory subunit 1B | NM_001113311 /// XM_001789781 | -2.502 | 2.07E-02 |
| Bt.24370.1.A1_at | *CKS2* | CDC28 protein kinase regulatory subunit 2 | NM_001113319 /// XM_001252074 | -4.013 | 5.16E-03 |

| | | | | | |
|---|---|---|---|---|---|
| Bt.393.1.S2_at | *CLDN11* | claudin 11 | NM_001035055 | 4.562 | 4.84E-02 |
| Bt.20428.2.S1_a_at | *CLDN5* | claudin 5 | NM_001076460 | 2.525 | 1.14E-02 |
| Bt.12916.1.S1_at | *CLU* | clusterin | NM_173902 | 2.062 | 2.69E-02 |
| Bt.1185.2.S1_at | *CNDP2* | CNDP dipeptidase 2 (metallopeptidase M20 family) | NM_001035108 | 2.025 | 6.27E-03 |
| Bt.986.1.A1_at | *COL4A6* | collagen, type IV, alpha 6 | XM_601826 | 2.091 | 3.12E-02 |
| Bt.26683.2.S1_at | *CORO1A* | coronin, actin binding protein, 1A | NM_174521 | 2.087 | 1.18E-02 |
| Bt.20784.1.A1_at | *CP* | ceruloplasmin (ferroxidase) | XM_592003 | 3.176 | 1.94E-02 |
| Bt.7331.1.S1_at | *CP* | ceruloplasmin (ferroxidase) | XM_592003 | 2.891 | 9.03E-03 |
| Bt.13289.2.S1_at | *CP* | ceruloplasmin (ferroxidase) | XM_592003 | 2.435 | 7.52E-03 |
| Bt.20836.1.S1_at | *CRYM* | crystallin, mu | NM_001045914 | -2.202 | 3.71E-02 |
| Bt.13413.2.A1_at | *CSTB* | cystatin B (stefin B) | NM_001100362 | 2.625 | 2.00E-02 |
| Bt.28225.2.S1_at | *CTSB* | cathepsin B | NM_174031 | 2.936 | 4.53E-03 |

| | | | | | |
|---|---|---|---|---|---|
| Bt.6800.1.A1_at | *CTSB* | cathepsin B | NM_174031 | 2.498 | 4.55E-03 |
| Bt.9807.1.S1_at | *CTSB* | Cathepsin B | NM_174031 | 2.345 | 4.53E-03 |
| Bt.27826.1.S1_at | *CTSF* | cathepsin F | NM_001075416 | 2.321 | 7.64E-03 |
| Bt.13834.1.S1_at | *CTSW* | cathepsin W | NM_001110070 | 2.221 | 2.29E-02 |
| Bt.284.1.S1_at | *CTSZ* | cathepsin Z | NM_001077835 | 2.103 | 5.16E-03 |
| Bt.9863.1.S1_at | *CXCL14* | chemokine (C-X-C motif) ligand 14 | NM_001034410 | -3.679 | 5.06E-03 |
| Bt.1185.1.A1_at | *CXCR4* | chemokine (C-X-C motif) receptor 4 | NM_174301 | 3.909 | 6.84E-03 |
| Bt.13387.1.S1_at | *CYB5R3* | cytochrome b5 reductase 3 | NM_001103250 | -2.165 | 5.02E-03 |
| Bt.28366.2.S1_at | *CYBB* | cytochrome b-245, beta polypeptide | NM_174035 | 2.200 | 4.91E-03 |
| Bt.16058.2.S1_at | *CYP11A1* | cytochrome P450, family 11, subfamily A, polypeptide 1 | NM_176644 | -2.485 | 1.17E-02 |
| Bt.14371.1.A1_at | *DCK* | deoxycytidine kinase | NM_001034573 | -2.349 | 2.59E-02 |
| Bt.16538.2.A1_at | *DCLK1* | Doublecortin-like kinase 1 | NM_001109962 | 2.357 | 4.91E-03 |

| | | | | | |
|---|---|---|---|---|---|
| Bt.27600.1.A1_at | *DDX39* | DEAD (Asp-Glu-Ala-Asp) box polypeptide 39 | NM_001034752 | -2.557 | 1.62E-02 |
| Bt.24218.1.S1_at | *DSN1* | DSN1, MIND kinetochore complex component, homolog (S. cerevisiae) | NM_001100323 | -3.164 | 1.51E-02 |
| Bt.22672.1.A1_at | *DTYMK* | deoxythymidylate kinase (thymidylate kinase) | NM_001098893 | -3.118 | 1.26E-02 |
| Bt.1920.2.S1_at | *DUSP26* | dual specificity phosphatase 26 (putative) | NM_001076004 | 2.339 | 4.55E-03 |
| Bt.5536.1.S1_at | *DUT* | deoxyuridine triphosphatase | NM_001077873 | -2.586 | 4.55E-03 |
| Bt.964.1.S1_at | *DUT* | deoxyuridine triphosphatase | NM_001077873 | -2.708 | 4.53E-03 |
| Bt.9807.2.S1_at | *DYNLRB2* | dynein, light chain, roadblock-type 2 | NM_001077119 | 2.343 | 5.99E-03 |
| Bt.18081.1.A1_at | *E2F8* | E2F transcription factor 8 | XM_001254260 | -3.813 | 9.95E-03 |
| Bt.1088.1.S1_a_at | *ECT2* | epithelial cell transforming sequence 2 oncogene | NM_001097573 | -3.206 | 7.36E-03 |
| Bt.28826.1.A1_at | *ECT2* | epithelial cell transforming sequence 2 oncogene | NM_001097573 | -3.508 | 5.16E-03 |
| Bt.3863.1.S1_at | *EGR1* | early growth response 1 | NM_001045875 | 2.297 | 2.71E-02 |
| Bt.1296.2.S1_at | *ENPP2* | ectonucleotide pyrophosphatase/phosphodiesterase 2 | NM_001080293 | 2.301 | 1.99E-02 |

| | | | | | |
|---|---|---|---|---|---|
| Bt.13476.1.S1_at | *EPHX2 /// LOC785508* | epoxide hydrolase 2, cytoplasmic /// similar to epoxide hydrolase 2, cytoplasmic | NM_001075534 /// XM_001253506 | 2.388 | 1.95E-02 |
| Bt.3371.1.S1_at | *ERCC6L* | excision repair cross-complementing rodent repair deficiency, complementation gr | NM_001102530 | -3.228 | 1.46E-02 |
| Bt.28243.2.S1_at | *ESPL1* | extra spindle pole bodies homolog 1 (S. cerevisiae) | NM_001045949 /// XM_001790117 | -5.224 | 5.24E-03 |
| Bt.12876.3.S1_a_at | *EZH2* | enhancer of zeste homolog 2 (Drosophila) | XM_585997 /// XM_865659 | -2.122 | 5.74E-03 |
| Bt.12295.1.S1_at | *FABP5* | fatty acid binding protein 5 (psoriasis-associated) | NM_174315 | 2.512 | 4.26E-02 |
| Bt.29850.1.S1_at | *FAM83D* | Protein FAM83D | NM_001083393 | -3.747 | 5.16E-03 |
| Bt.7058.1.S1_at | *FAM84A* | family with sequence similarity 84, member A | NM_001034659 | 2.053 | 5.24E-03 |
| Bt.13865.1.A1_at | *FANCI* | Fanconi anemia, complementation group I | XM_600723 | -3.179 | 1.30E-02 |
| Bt.1742.2.S1_a_at | *FBXO32* | F-box protein 32 | NM_001046155 | 2.640 | 2.20E-02 |
| Bt.4995.1.A1_at | *FBXO32* | F-box protein 32 | NM_001046155 | 2.102 | 4.10E-02 |
| Bt.5373.1.S1_at | *FBXO5* | F-box protein 5 | NM_001082435 | -3.238 | 9.90E-03 |
| Bt.24600.1.S1_at | *FCER1A* | Fc fragment of IgE, high affinity I, receptor for; alpha polypeptide | NM_001100310 | 2.073 | 5.24E-03 |

| | | | | | |
|---|---|---|---|---|---|
| Bt.6687.1.S1_at | *FCGR1A* | Fc fragment of IgG, high affinity Ia, receptor (CD64) | NM_174538 | 2.470 | 9.27E-03 |
| Bt.20677.1.S1_at | *FEN1* | flap structure-specific endonuclease 1 | NM_001035113 | -2.876 | 1.29E-02 |
| Bt.28799.1.S1_at | *FERMT3* | fermitin family homolog 3 (Drosophila) | NM_001037606 | 2.220 | 6.18E-03 |
| Bt.20262.1.S1_at | *FGFR2* | fibroblast growth factor receptor 2 | XM_001789706 | -2.251 | 1.59E-02 |
| Bt.26683.1.S1_at | *FGL1* | fibrinogen-like 1 | NM_001034313 | 2.259 | 8.70E-03 |
| Bt.12744.1.S1_at | *FGL2* | fibrinogen-like 2 | NM_001046097 | 2.731 | 1.48E-02 |
| Bt.8718.1.S1_at | *FOLR2* | folate receptor 2 (fetal) | NM_001075325 | 3.294 | 4.91E-03 |
| Bt.28225.1.A1_at | *FOS* | FBJ murine osteosarcoma viral oncogene homolog | NM_182786 | 3.101 | 3.25E-02 |
| Bt.17915.1.S1_at | *FOS* | FBJ murine osteosarcoma viral oncogene homolog | NM_182786 | 3.083 | 4.14E-02 |
| Bt.25478.1.A1_at | *FOSL2* | FOS-like antigen 2 | XM_864138 | 3.101 | 6.90E-03 |
| Bt.15682.1.A1_at | *FOXM1* | forkhead box M1 | XM_001249704 /// XM_872119 | -2.082 | 1.56E-02 |
| Bt.28409.1.A1_at | *GABARAPL1* | GABA(A) receptor-associated protein like 1 | NM_001033616 | 2.048 | 8.94E-03 |

| | | | | | |
|---|---|---|---|---|---|
| Bt.20431.1.A1_at | *GAMT* | guanidinoacetate N-methyltransferase | NM_001038544 | 2.058 | 2.33E-02 |
| Bt.6406.1.S3_at | *GAS7* | growth arrest-specific 7 | NM_001102280 | 2.086 | 7.74E-03 |
| Bt.1220.1.S1_at | *GEM* | GTP binding protein overexpressed in skeletal muscle | NM_001083732 | 2.248 | 2.05E-02 |
| Bt.13336.2.S1_at | *GIMAP7* | GTPase, IMAP family member 7 | NM_001080257 | 2.451 | 2.54E-02 |
| Bt.2520.1.S1_at | *GIMAP7* | GTPase, IMAP family member 7 | NM_001080257 | 2.263 | 3.09E-02 |
| Bt.7620.1.S1_at | *GINS3* | GINS complex subunit 3 (Psf3 homolog) | NM_001076136 | -2.142 | 8.03E-03 |
| Bt.12961.1.A1_at | *GINS3* | GINS complex subunit 3 (Psf3 homolog) | NM_001076136 | -2.165 | 1.33E-02 |
| Bt.10510.1.S1_at | *GMFG* | glia maturation factor, gamma | NM_001024537 | 2.137 | 6.18E-03 |
| Bt.4817.1.A1_at | *GPNMB* | glycoprotein (transmembrane) nmb | NM_001038065 | 14.505 | 6.44E-03 |
| Bt.11936.1.S1_at | *GPNMB* | glycoprotein (transmembrane) nmb | NM_001038065 | 3.906 | 7.41E-03 |
| Bt.26415.1.A1_at | *GPNMB* | glycoprotein (transmembrane) nmb | NM_001038065 | 2.139 | 1.84E-02 |
| Bt.6397.1.S1_at | *GPR77* | G protein-coupled receptor 77 | NM_001077947 | 2.265 | 4.04E-02 |

| | | | | | |
|---|---|---|---|---|---|
| Bt.17488.1.A1_at | *GPX3* | glutathione peroxidase 3 (plasma) | NM_174077 | 3.394 | 6.17E-03 |
| Bt.2111.1.S1_a_at | *H2AFX* | H2A histone family, member X | NM_001079780 | -2.831 | 8.94E-03 |
| Bt.21546.1.S1_at | *H2AFZ* | H2A histone family, member Z | NM_174809 | -2.029 | 1.61E-02 |
| Bt.7035.1.S1_at | *HJURP* | Holliday junction recognition protein | XM_869720 | -5.629 | 4.91E-03 |
| Bt.15733.1.A1_at | *HMGB2* | High-mobility group box 2 | NM_001037616 | -2.001 | 6.81E-03 |
| Bt.11497.1.A1_at | *HMGB2* | high-mobility group box 2 | NM_001037616 | -2.345 | 5.49E-03 |
| Bt.20499.1.S1_at | *HMMR* | hyaluronan-mediated motility receptor (RHAMM) | XM_590028 | -2.058 | 1.33E-02 |
| Bt.19819.1.S1_at | *HMMR* | hyaluronan-mediated motility receptor (RHAMM) | XM_590028 | -3.575 | 5.84E-03 |
| Bt.1343.2.A1_at | *HMOX1* | heme oxygenase (decycling) 1 | NM_001014912 | 2.378 | 8.78E-03 |
| Bt.21939.1.S1_at | *HN1* | hematological and neurological expressed 1 | NM_001034734 | -2.315 | 1.43E-02 |
| Bt.9155.1.A1_at | *HPGD* | hydroxyprostaglandin dehydrogenase 15-(NAD) | NM_001034419 | -2.402 | 2.10E-02 |
| Bt.2556.1.S1_at | *HPGD* | hydroxyprostaglandin dehydrogenase 15-(NAD) | NM_001034419 | -3.001 | 2.09E-02 |

| | | | | | |
|---|---|---|---|---|---|
| Bt.9997.1.S1_at | *HRH1* | histamine receptor H1 | NM_174083 | 2.068 | 6.84E-03 |
| Bt.3560.1.S1_at | *HSD17B11* | hydroxysteroid (17-beta) dehydrogenase 11 | NM_001046286 | 2.417 | 1.24E-02 |
| Bt.2410.1.A1_at | *IL1R1* | interleukin 1 receptor, type I | XM_593695 | 2.265 | 4.55E-03 |
| Bt.5193.1.S1_at | *IL6* | interleukin 6 (interferon, beta 2) | NM_173923 | 2.630 | 2.38E-02 |
| Bt.15739.1.S1_at | *IQCA1* | IQ motif containing with AAA domain 1 | XM_001790491 /// XM_870442 | 2.080 | 5.16E-03 |
| Bt.17513.1.A1_at | *IQGAP3* | IQ motif containing GTPase activating protein 3 | NM_001098880 | -2.691 | 5.49E-03 |
| Bt.2334.1.S1_at | *ISM1* | isthmin 1 homolog (zebrafish) | NM_001163935 /// XM_583413 | 2.799 | 2.27E-02 |
| Bt.6456.1.A1_at | *ITGB2* | integrin, beta 2 (complement component 3 receptor 3 and 4 subunit) | NM_175781 | 2.152 | 7.59E-03 |
| Bt.10816.1.S1_s_at | *ITGB5* | integrin, beta 5 | NM_174679 | 2.216 | 2.78E-02 |
| Bt.26660.1.S1_at | *JUN* | jun oncogene | NM_001077827 | 2.609 | 1.51E-02 |
| Bt.22083.2.S1_at | *JUN* | jun oncogene | NM_001077827 | 2.232 | 2.69E-02 |
| Bt.28037.1.S1_at | *KANK1* | KN motif and ankyrin repeat domains 1 | XM_614789 | 2.009 | 1.99E-02 |

| | | | | | |
|---|---|---|---|---|---|
| Bt.22460.1.S2_at | *KCNMA1* | potassium large conductance calcium-activated channel, subfamily M, alpha member | NM_174680 | -3.274 | 2.22E-02 |
| Bt.4631.2.S1_at | *KIF20A* | kinesin family member 20A | NM_001046288 | -3.719 | 4.94E-03 |
| Bt.278.1.S1_at | *KIF20A* | kinesin family member 20A | NM_001046288 | -5.109 | 5.24E-03 |
| Bt.10253.1.A1_at | *KIF22* | kinesin family member 22 | NM_001101868 | -3.505 | 5.26E-03 |
| Bt.19274.1.A1_at | *KIF23* | kinesin family member 23 | NM_001098038 | -3.013 | 5.84E-03 |
| Bt.26952.1.S1_at | *KIF2C* | kinesin family member 2C | NM_001101147 | -2.232 | 5.53E-03 |
| Bt.22869.1.S1_at | *KIF2C* | kinesin family member 2C | NM_001101147 | -5.024 | 6.17E-03 |
| Bt.1978.1.S1_at | *KIF4A* | kinesin family member 4A | XM_617283 | -3.753 | 5.31E-03 |
| Bt.2353.1.S1_at | *KIFC1* | kinesin family member C1 | NM_001101936 | -3.286 | 5.49E-03 |
| Bt.351.1.S1_at | *KLF15* | Kruppel-like factor 15 | NM_001082425 | 2.106 | 1.37E-02 |
| Bt.17821.3.A1_at | *KLHL24* | kelch-like 24 (Drosophila) | XM_866793 | 2.098 | 3.75E-02 |
| Bt.6441.1.S1_at | *KNTC1* | kinetochore associated 1 | XM_582786 | -3.620 | 8.11E-03 |

| | | | | | |
|---|---|---|---|---|---|
| Bt.12504.1.S1_at | *KPNA2* | karyopherin alpha 2 (RAG cohort 1, importin alpha 1) | NM_001034449 | -3.723 | 5.16E-03 |
| Bt.13975.2.A1_at | *LAPTM5* | lysosomal protein transmembrane 5 | NM_001046118 | 2.369 | 6.94E-03 |
| Bt.5193.2.S1_a_at | *LCP1* | lymphocyte cytosolic protein 1 (L-plastin) | NM_001034720 | 2.533 | 4.91E-03 |
| Bt.12217.1.S1_at | *LGALS3* | lectin, galactoside-binding, soluble, 3 | NM_001102341 | 4.954 | 4.91E-03 |
| Bt.9807.3.S1_at | *LGMN* | legumain | NM_174101 | 2.010 | 5.06E-03 |
| Bt.2515.1.S1_at | *LIG1* | ligase I, DNA, ATP-dependent | NM_001102548 | -2.004 | 1.43E-02 |
| Bt.28346.2.S1_a_at | *LIPG* | lipase, endothelial | XM_586851 | -2.201 | 3.34E-02 |
| Bt.2529.1.S1_at | *LMNB1* | lamin B1 | NM_001103295 | -3.670 | 6.90E-03 |
| Bt.23106.1.S1_at | *LOC100138846 /// LOC784932 /// LOC784964 /// SERPINA3 /// SERPINA3-1 /// SERPINA3-2 /// SERPINA3-7* | similar to endopin 2C /// similar to endopin 2B /// similar to Endopin 1b /// se | NM_001012283 /// NM_001081744 /// NM_001146301 /// NM_174768 /// XM_001252646 // | -2.137 | 4.75E-02 |
| Bt.28430.1.S1_at | *LOC100139092* | similar to Probable G-protein coupled receptor 34 | XR_042936 | 2.090 | 2.12E-02 |

| | | | | | |
|---|---|---|---|---|---|
| Bt.3681.1.S1_at | *LOC100139766 /// LOC507024 /// LOC510212 /// LOC618416 /// LOC783134* | similar to immunoglobulin-like transcript 5 protein /// similar to Leukocyte imm | XM_001787794 /// XM_001788788 /// XM_583568 /// XR_042676 /// XR_042799 | 2.159 | 9.69E-03 |
| Bt.21159.1.S1_at | *LOC100302527* | hypothetical protein LOC100302527 | NM_001163442 | -2.066 | 7.71E-03 |
| Bt.26604.2.S1_at | *LOC504773* | regakine 1 | NM_001034220 | 4.946 | 4.91E-03 |
| Bt.28741.2.S1_at | *LOC507141* | CE5 protein-like | XM_583699 | 2.298 | 2.03E-02 |
| Bt.10391.1.S1_at | *LOC509513 /// TRB@* | hypothetical LOC509513 /// T cell receptor, beta cluster | NM_001080904 | 2.213 | 3.20E-02 |
| Bt.23779.1.A1_at | *LOC513508* | similar to acyl-Coenzyme A dehydrogenase family, member 10 | XM_001788744 | 2.123 | 2.61E-02 |
| Bt.1440.1.S1_at | *LOC513587* | Similar to UPF0474 protein C5orf41 | XM_876203 | 2.463 | 5.53E-03 |
| Bt.26046.1.A1_at | *LOC513587* | Similar to UPF0474 protein C5orf41 | XM_876203 | 2.101 | 8.57E-03 |
| Bt.22120.1.S1_at | *LOC516579* | similar to ATPase, Class II, type 9A | XR_028542 | 2.519 | 1.31E-02 |
| Bt.13458.1.S1_at | *LOC518821* | similar to Centromeric protein E (CENP-E) | XR_027308 | -4.967 | 4.91E-03 |
| Bt.3017.1.S1_at | *LOC521099* | similar to family with sequence similarity 20, member A | XM_599356 | 2.025 | 1.63E-02 |

| | | | | | |
|---|---|---|---|---|---|
| Bt.11525.2.A1_at | *LOC529947* | similar to signal peptide, CUB domain, EGF-like 2 | XM_608409 | 3.166 | 5.24E-03 |
| Bt.28162.3.S1_at | *LOC530077* | similar to GTPase, IMAP family member 5 | XM_864682 | 2.181 | 1.41E-02 |
| Bt.25773.1.A1_at | *LOC535166* | similar to mKIAA1077 protein | XR_027912 | 2.621 | 1.13E-02 |
| Bt.21087.1.A1_at | *LOC539596* | similar to Transforming growth factor-beta induced protein IG-H3 precursor (Beta | XR_028016 | 2.121 | 5.68E-03 |
| Bt.1296.1.S1_at | *LOC540455* | hypothetical protein LOC540455 | NM_001099109 | -7.261 | 4.91E-03 |
| Bt.15856.1.S1_at | *LOC614351* | similar to DNA replication complex GINS protein PSF2 | XM_865829 | -2.170 | 1.58E-02 |
| Bt.26968.1.S1_at | *LOC615685* | UPF0452 protein C7orf41 homolog | NM_001101246 | 2.063 | 9.41E-03 |
| Bt.14166.2.S1_at | *LOC617744* | hypothetical LOC617744 | XR_043042 | 2.202 | 1.24E-02 |
| Bt.24411.1.A1_at | *LOC618360* | similar to Nitric oxide-inducible gene protein | XM_870692 | -2.261 | 2.07E-02 |
| Bt.22390.2.S1_at | *LOC618591* | similar to NKp80 NK receptor | NM_001101276 | 2.249 | 4.91E-03 |
| Bt.10236.1.S1_at | *LOC782922* | similar to prostaglandin F synthetase II | NM_001166224 /// XM_001250622 | 2.216 | 9.48E-03 |
| Bt.20074.1.S1_a_at | *LOC783399* | major allergen Equ c 1-like | XM_001252015 | 3.292 | 1.65E-02 |

| | | | | | |
|---|---|---|---|---|---|
| Bt.26604.1.S1_at | *LOC784007* | Similar to LOC496253 protein | XM_001252441 | 2.986 | 5.35E-03 |
| Bt.20570.1.S1_at | *LOC784034* | similar to Uncharacterized protein C1orf112 homolog | XM_001252463 | -2.371 | 1.95E-02 |
| Bt.15587.1.S1_at | *LSM4* | LSM4 homolog, U6 small nuclear RNA associated (S. cerevisiae) | NM_001035436 | -2.010 | 1.38E-02 |
| Bt.24813.1.A1_at | *LTF* | lactotransferrin | NM_180998 | 2.303 | 2.95E-02 |
| Bt.14379.2.S1_at | *LY9* | lymphocyte antigen 9 | NM_001075918 | 2.179 | 1.03E-02 |
| Bt.20778.3.A1_at | *MAD2 /// MAD2L1* | mitotic checkpoint component Mad2 /// MAD2 mitotic arrest deficient-like 1 (yeas | NM_001079796 /// XM_001254565 | -3.409 | 1.09E-02 |
| Bt.1929.1.S1_at | *MAGOHB* | mago-nashi homolog B (Drosophila) | NM_001076252 | -2.096 | 7.45E-03 |
| Bt.5819.1.S1_at | *MANF* | mesencephalic astrocyte-derived neurotrophic factor | NM_001101211 | -2.191 | 6.90E-03 |
| Bt.13413.1.S1_at | *MAOB* | monoamine oxidase B | NM_177944 | 2.567 | 2.33E-02 |
| Bt.858.1.S1_at | *MCM2* | similar to KIAA0030 | XM_864352 | -2.501 | 2.94E-02 |
| Bt.27090.1.S1_at | *MCM3* | minichromosome maintenance complex component 3 | NM_001013586 | -3.118 | 2.43E-02 |
| Bt.25834.1.A1_at | *MCM4* | minichromosome maintenance complex component 4 | NM_001075158 | -3.387 | 1.63E-02 |

| | | | | | |
|---|---|---|---|---|---|
| Bt.21607.1.S1_at | *MCM5* | minichromosome maintenance complex component 5 | NM_001075290 | -2.408 | 1.40E-02 |
| Bt.13480.1.S1_at | *MCM6* | minichromosome maintenance complex component 6 | NM_001046234 | -2.159 | 2.45E-02 |
| Bt.3804.1.S1_at | *MELK* | maternal embryonic leucine zipper kinase | NM_001111260 /// XM_001787811 | -4.538 | 7.85E-03 |
| Bt.639.1.S1_at | *MERTK* | c-mer proto-oncogene tyrosine kinase | XM_580552 | 2.767 | 4.53E-03 |
| Bt.1416.1.S1_at | *MMP19* | matrix metallopeptidase 19 | NM_001075983 | 2.974 | 8.58E-03 |
| Bt.21408.1.S1_at | *MOBKL2B* | MOB1, Mps One Binder kinase activator-like 2B (yeast) | NM_001046491 | 2.186 | 1.68E-02 |
| Bt.5993.1.S1_at | *MPEG1* | macrophage expressed 1 | NM_001046464 | 2.591 | 5.16E-03 |
| Bt.22117.1.S1_at | *MRC1L1* | mannose receptor, C type 1-like 1 | XM_001252128 | 2.500 | 5.88E-03 |
| Bt.6537.1.S1_at | *MRC1L1* | mannose receptor, C type 1-like 1 | XM_001252128 | 2.419 | 1.46E-02 |
| Bt.20574.1.S1_at | *MRPL15* | mitochondrial ribosomal protein L15 | NM_001075445 | -2.010 | 2.41E-02 |
| Bt.12217.2.S1_at | *MSR1* | macrophage scavenger receptor 1 | NM_001113240 /// NM_174113 | 3.375 | 4.91E-03 |
| Bt.17179.1.S1_at | *MT2A* | metallothionein 2A | NM_001075140 /// XM_586929 | 2.740 | 2.73E-02 |

| | | | | | |
|---|---|---|---|---|---|
| Bt.7670.1.A1_at | *MTHFD1* | methylenetetrahydrofolate dehydrogenase (NADP+ dependent) 1, methenyltetrahydrof | NM_001083477 | -2.034 | 1.24E-02 |
| Bt.28142.1.S1_at | *MXD3* | MAX dimerization protein 3 | XM_883278 | -3.018 | 4.91E-03 |
| Bt.29925.1.S1_at | *MXI1* | MAX interactor 1 | NM_001076331 | 2.073 | 1.32E-02 |
| Bt.27319.2.S1_at | *MXI1* | MAX interactor 1 | NM_001076331 | 2.027 | 9.97E-03 |
| Bt.29923.1.S1_at | *MXRA8* | matrix-remodelling associated 8 | NM_001075830 | 2.511 | 9.50E-03 |
| Bt.8803.1.A1_at | *MYBL2* | v-myb myeloblastosis viral oncogene homolog (avian)-like 2 | NM_001075448 | -2.117 | 7.58E-03 |
| Bt.2806.1.S1_at | *MYH11* | myosin, heavy chain 11, smooth muscle | NM_001102127 | 2.160 | 4.77E-02 |
| Bt.1577.1.S1_at | *N4BP2L1* | NEDD4 binding protein 2-like 1 | NM_001046574 | 2.222 | 1.80E-02 |
| Bt.28266.1.A1_at | *NCAPD3* | non-SMC condensin II complex, subunit D3 | XM_868423 | -2.036 | 1.16E-02 |
| Bt.6129.1.S1_at | *NCAPG* | non-SMC condensin I complex, subunit G | NM_001102376 | -3.984 | 4.91E-03 |
| Bt.11129.1.S1_at | *NCAPG2* | non-SMC condensin II complex, subunit G2 | XM_594801 | -3.184 | 1.73E-02 |
| Bt.28960.1.A1_at | *NCAPH* | non-SMC condensin I complex, subunit H | NM_001034213 | -2.104 | 1.52E-02 |

| | | | | | |
|---|---|---|---|---|---|
| Bt.28797.1.A1_at | *NCAPH* | non-SMC condensin I complex, subunit H | NM_001034213 | -4.593 | 5.90E-03 |
| Bt.2913.1.A1_at | *NDC80* | NDC80 homolog, kinetochore complex component (S. cerevisiae) | XM_582722 | -4.151 | 5.98E-03 |
| Bt.7035.2.S1_at | *NDRG1* | N-myc downstream regulated 1 | NM_001035009 | 2.214 | 4.91E-03 |
| Bt.13330.2.A1_at | *NKG7* | natural killer cell group 7 sequence | NM_001035279 | 2.157 | 4.91E-03 |
| Bt.20074.2.S1_at | *NOL3* | nucleolar protein 3 (apoptosis repressor with CARD domain) | NM_001083688 | 2.950 | 4.55E-03 |
| Bt.22759.1.S1_at | *NOL3* | nucleolar protein 3 (apoptosis repressor with CARD domain) | NM_001083688 | 2.038 | 1.05E-02 |
| Bt.9538.1.S1_at | *NPL* | N-acetylneuraminate pyruvate lyase (dihydrodipicolinate synthase) | NM_001045978 | 2.238 | 4.66E-03 |
| Bt.4374.1.S1_at | *NRM* | nurim (nuclear envelope membrane protein) | NM_001037596 | -2.223 | 4.91E-03 |
| Bt.28502.1.S1_at | *NRP2* | neuropilin 2 | XM_614062 /// XM_865994 /// XM_877502 | 2.594 | 4.91E-03 |
| Bt.4670.1.S1_at | *NRP2* | neuropilin 2 | XM_614062 /// XM_865994 /// XM_877502 | 2.489 | 1.17E-02 |
| Bt.4010.1.S1_at | *NRP2* | neuropilin 2 | XM_614062 /// XM_865994 /// XM_877502 | 2.083 | 7.71E-03 |
| Bt.14005.1.S1_at | *NSL1* | NSL1, MIND kinetochore complex component, homolog (S. cerevisiae) | NM_001101876 | -2.002 | 2.73E-02 |

| | | | | | |
|---|---|---|---|---|---|
| Bt.12217.3.A1_at | *NTRK2* | Neurotrophic tyrosine kinase, receptor, type 2 | NM_001075225 | 4.448 | 3.11E-02 |
| Bt.22430.1.A1_at | *NTRK2* | Neurotrophic tyrosine kinase, receptor, type 2 | NM_001075225 | 3.828 | 3.02E-02 |
| Bt.4902.1.S1_at | *NTRK2* | neurotrophic tyrosine kinase, receptor, type 2 | NM_001075225 | 2.730 | 2.07E-02 |
| Bt.26155.1.A1_at | *NUPR1* | nuclear protein, transcriptional regulator, 1 | NM_001114515 /// XM_867457 | 3.544 | 6.27E-03 |
| Bt.27531.1.A1_at | *NUSAP1* | nucleolar and spindle associated protein 1 | NM_001046571 | -5.067 | 5.06E-03 |
| Bt.24844.1.S1_at | *OIP5* | Opa interacting protein 5 | XM_588370 | -3.765 | 8.94E-03 |
| Bt.20397.1.S1_at | *OIT3* | oncoprotein induced transcript 3 | NM_001046064 | -3.856 | 5.16E-03 |
| Bt.26309.1.A1_at | *ORC1L* | origin recognition complex, subunit 1-like (yeast) | NM_001014918 | -2.617 | 2.18E-02 |
| Bt.21343.2.A1_a_at | *OSMR* | oncostatin M receptor | NM_001080272 | 2.378 | 1.54E-02 |
| Bt.23089.1.S1_at | *OSMR* | oncostatin M receptor | NM_001080272 | 2.342 | 1.24E-02 |
| Bt.26855.1.S1_at | *PAF* | KIAA0101 protein | NM_001015678 | -6.987 | 5.24E-03 |
| Bt.3537.1.S1_at | *PARP12* | poly (ADP-ribose) polymerase family, member 12 | XM_590833 | 2.033 | 4.55E-03 |

| | | | | | |
|---|---|---|---|---|---|
| Bt.5278.2.S1_at | *PCDH7* | protocadherin 7 | XM_864701 | -2.115 | 3.38E-02 |
| Bt.28062.1.S1_at | *PCNA* | proliferating cell nuclear antigen | NM_001034494 | -2.596 | 1.58E-02 |
| Bt.12606.1.S1_a_at | *PCOLCE2* | procollagen C-endopeptidase enhancer 2 | NM_001075629 | 2.143 | 3.25E-02 |
| Bt.26890.1.S1_at | *PDK4* | pyruvate dehydrogenase kinase, isozyme 4 | NM_001101883 | 2.200 | 9.16E-03 |
| Bt.16687.1.A1_at | *PDK4* | pyruvate dehydrogenase kinase, isozyme 4 | NM_001101883 | 2.045 | 8.36E-03 |
| Bt.1343.1.S1_at | *PDPN* | podoplanin | NM_001033120 | 2.138 | 5.84E-03 |
| Bt.3532.1.A1_at | *PHGDH* | phosphoglycerate dehydrogenase | NM_001035017 | -2.659 | 5.35E-03 |
| Bt.22969.1.S1_at | *PID1* | phosphotyrosine interaction domain containing 1 | NM_001079584 | 2.993 | 9.50E-03 |
| Bt.16725.1.S1_at | *PKIB* | protein kinase (cAMP-dependent, catalytic) inhibitor beta | NM_001114518 /// XM_001788472 /// XM_001788483 /// XM_001788486 /// XM_001788495 | 4.523 | 6.90E-03 |
| Bt.41.1.S1_at | *PLA1A* | phospholipase A1 member A | NM_001015610 | 2.801 | 4.91E-03 |
| Bt.22385.2.S1_a_at | *PLA1A* | phospholipase A1 member A | NM_001015610 | 2.354 | 4.91E-03 |

| | | | | | |
|---|---|---|---|---|---|
| Bt.13583.1.A1_at | *PLIN5* | perilipin 5 | NM_001101136 | -2.209 | 1.94E-02 |
| Bt.14219.1.S1_at | *PLK1* | polo-like kinase 1 (Drosophila) | NM_001038173 | -2.332 | 7.87E-03 |
| Bt.23094.4.S1_at | *PLN* | phospholamban | NM_001103319 | 2.174 | 5.02E-03 |
| Bt.13330.1.S1_at | *PLXNC1* | plexin C1 | XM_596354 | 2.148 | 1.00E-02 |
| Bt.4995.2.S1_at | *POLE2* | polymerase (DNA directed), epsilon 2 (p59 subunit) | NM_001105383 | -2.562 | 2.69E-02 |
| Bt.3734.1.A1_at | *PPA1* | pyrophosphatase (inorganic) 1 | NM_001075118 | -2.059 | 2.79E-02 |
| Bt.12087.1.A1_at | *PRC1* | protein regulator of cytokinesis 1 | NM_001076543 | -4.719 | 5.24E-03 |
| Bt.864.2.S1_at | *PRDM1* | PR domain containing 1, with ZNF domain | XM_618588 | 2.180 | 5.45E-03 |
| Bt.4482.1.S1_at | *PSAT1* | phosphoserine aminotransferase 1 | NM_001102150 | -2.096 | 1.44E-02 |
| Bt.15725.1.A1_at | *PSRC1* | proline/serine-rich coiled-coil 1 | NM_001046501 | -2.254 | 7.17E-03 |
| Bt.18316.2.S1_at | *PTGDS* | prostaglandin D2 synthase 21kDa (brain) | NM_174791 | 2.199 | 2.04E-02 |
| Bt.23668.1.A1_at | *PTGER4* | prostaglandin E receptor 4 (subtype EP4) | NM_174589 | 2.548 | 9.71E-03 |

| | | | | | |
|---|---|---|---|---|---|
| Bt.18776.1.S1_at | *PTTG1* | pituitary tumor-transforming 1 | NM_001034310 | -6.471 | 5.16E-03 |
| Bt.13134.1.S1_at | *RACGAP1* | Rac GTPase activating protein 1 | XM_592496 | -3.196 | 5.41E-03 |
| Bt.25412.1.A1_at | *RAD51AP1* | RAD51 associated protein 1 | NM_001076403 | -5.508 | 7.64E-03 |
| Bt.8939.1.S1_at | *RASGEF1B* | RasGEF domain family, member 1B | NM_001083649 | 2.664 | 5.99E-03 |
| Bt.27087.1.A1_at | *RBM5* | RNA binding motif protein 5 | NM_001046374 | 2.686 | 3.63E-02 |
| Bt.18557.1.S1_at | *RBPMS* | RNA binding protein with multiple splicing | NM_001046535 | 2.299 | 4.83E-03 |
| Bt.5506.1.A1_at | *RDM1* | RAD52 motif 1 | XM_591173 | -2.110 | 1.79E-02 |
| Bt.29403.1.S1_at | *RENBP* | renin binding protein | NM_001046223 /// XM_594363 | 2.223 | 7.64E-03 |
| Bt.19884.1.A1_at | *RGS1* | regulator of G-protein signaling 1 | XM_001252887 | 3.006 | 3.63E-02 |
| Bt.6980.1.S1_at | *RGS2* | regulator of G-protein signaling 2, 24kDa | NM_001075596 | 2.671 | 1.76E-02 |
| Bt.27645.1.A1_at | *RMI2* | chromosome 16 open reading frame 75 ortholog | NM_001099720 | -2.718 | 2.29E-02 |
| Bt.6406.1.S2_at | *RNASE6* | ribonuclease, RNase A family, k6 | NM_174594 | 2.031 | 2.64E-02 |

| | | | | | |
|---|---|---|---|---|---|
| Bt.20027.1.S1_at | *RPA2* | replication protein A2, 32kDa | NM_001045984 | -2.017 | 4.18E-02 |
| Bt.19567.2.S1_at | *RPA3* | replication protein A3, 14kDa | NM_001034427 | -2.309 | 6.27E-03 |
| Bt.26507.1.S1_at | *RRAD* | Ras-related associated with diabetes | NM_001045913 | 2.171 | 2.20E-02 |
| Bt.24859.1.A1_at | *RRM1* | ribonucleotide reductase M1 | NM_001113296 /// XM_879432 | -2.021 | 2.61E-02 |
| Bt.20277.1.S1_at | *RRM2* | ribonucleotide reductase M2 polypeptide | XM_584910 /// XM_875690 | -5.660 | 7.87E-03 |
| Bt.2113.1.S1_at | *RTP4* | receptor (chemosensory) transporter protein 4 | NM_001075961 | 2.302 | 4.55E-03 |
| Bt.16049.1.S1_at | *RYR2* | ryanodine receptor 2 (cardiac) | XM_001788290 | 2.410 | 3.25E-02 |
| Bt.8633.1.A1_at | *S100A13* | 8KDa amlexanox-binding protein | NM_205800 | 2.527 | 6.41E-03 |
| Bt.7467.1.S1_at | *S100A2* | S100 calcium binding protein A2 | NM_001034367 | -2.254 | 5.74E-03 |
| Bt.27140.1.S1_at | *SAA3* | serum amyloid A 3 | NM_181016 | 5.852 | 4.16E-02 |
| Bt.12328.1.S1_at | *SAMSN1* | SAM domain, SH3 domain and nuclear localization signals 1 | NM_001035404 | 2.855 | 4.83E-03 |
| Bt.24258.1.S1_at | *SAMSN1* | SAM domain, SH3 domain and nuclear localization signals 1 | NM_001035404 | 2.476 | 4.91E-03 |

| | | | | | |
|---|---|---|---|---|---|
| Bt.1377.1.S1_at | *SERPING1* | serpin peptidase inhibitor, clade G (C1 inhibitor), member 1 | NM_174821 | 3.931 | 4.91E-03 |
| Bt.393.1.S1_at | *SERPING1* | serpin peptidase inhibitor, clade G (C1 inhibitor), member 1 | NM_174821 | 3.662 | 4.91E-03 |
| Bt.7962.1.S1_at | *SFRS1* | splicing factor, arginine/serine-rich 1 | NM_001076394 | -2.294 | 7.57E-03 |
| Bt.7870.1.S1_at | *SGOL1* | shugoshin-like 1 (S. pombe) | NM_001081617 | -3.793 | 7.72E-03 |
| Bt.3756.1.A1_at | *SHCBP1* | SHC SH2-domain binding protein 1 | XM_601664 | -5.840 | 5.24E-03 |
| Bt.15231.1.S1_at | *SHISA2* | shisa homolog 2 (Xenopus laevis) | NM_001101265 | 2.184 | 3.46E-02 |
| Bt.6038.1.S1_at | *SIGLEC1* | sialic acid binding Ig-like lectin 1, sialoadhesin | XM_870818 | 2.234 | 7.41E-03 |
| Bt.27212.1.A1_at | *SKA1* | spindle and kinetochore associated complex subunit 1 | NM_001075327 | -4.355 | 6.57E-03 |
| Bt.1645.1.S1_at | *SKA3* | spindle and kinetochore associated complex subunit 3 | NM_001110067 | -2.962 | 1.95E-02 |
| Bt.1088.2.S1_at | *SKA3* | spindle and kinetochore associated complex subunit 3 | NM_001110067 | -3.281 | 9.29E-03 |
| Bt.6817.1.S1_at | *SLAMF7* | SLAM family member 7 | XM_001790094 | 2.676 | 5.24E-03 |
| Bt.27837.1.S1_at | *SLAMF9* | SLAM family member 9 | XM_864973 | 2.077 | 4.55E-03 |

| | | | | | |
|---|---|---|---|---|---|
| Bt.25039.1.S1_at | *SLC11A1* | solute carrier family 11 (proton-coupled divalent metal ion transporters), membe | NM_174652 | 2.189 | 2.54E-02 |
| Bt.15731.1.A1_at | *SLC16A1* | solute carrier family 16, member 1 (monocarboxylic acid transporter 1) | NM_001037319 | -2.264 | 1.79E-02 |
| Bt.3100.1.A1_at | *SLC24A6* | solute carrier family 24 (sodium/potassium/calcium exchanger), member 6 | NM_001177596 /// XM_585733 | 2.045 | 5.81E-03 |
| Bt.7264.1.S1_at | *SLC31A2* | solute carrier family 31 (copper transporters), member 2 | NM_001034556 | 2.177 | 4.55E-03 |
| Bt.3211.1.S2_at | *SLC40A1* | solute carrier family 40 (iron-regulated transporter), member 1 | NM_001077970 | 2.123 | 1.04E-02 |
| Bt.6397.2.S1_at | *SLC7A7* | solute carrier family 7 (cationic amino acid transporter, y+ system), member 7 | NM_001075151 | 2.650 | 5.16E-03 |
| Bt.16538.1.S1_at | *SLCO2A1* | solute carrier organic anion transporter family, member 2A1 | NM_174829 | -2.210 | 7.87E-03 |
| Bt.13537.1.A1_at | *SMC2* | structural maintenance of chromosomes 2 | XM_585705 | -2.504 | 3.59E-02 |
| Bt.15534.1.S1_a_at | *SMC4* | structural maintenance of chromosomes 4 | NM_001105326 | -2.313 | 6.57E-03 |
| Bt.1920.1.S1_at | *SMOC2* | SPARC related modular calcium binding 2 | NM_001098134 | 2.585 | 2.08E-02 |
| Bt.15730.1.S1_at | *SMOC2* | SPARC related modular calcium binding 2 | NM_001098134 | 2.306 | 1.96E-02 |

| | | | | | |
|---|---|---|---|---|---|
| Bt.20431.2.S1_at | *SNHG3-RCC1* | SNHG3-RCC1 readthrough transcript | NM_001105408 | -2.064 | 1.02E-02 |
| Bt.9517.1.S1_a_at | *SNX31* | sorting nexin 31 | XM_001249966 | 2.493 | 1.82E-02 |
| Bt.10696.1.S1_at | *SPAG5* | sperm associated antigen 5 | XM_580731 | -4.797 | 4.91E-03 |
| Bt.12236.1.S1_at | *SPC24* | SPC24, NDC80 kinetochore complex component, homolog (S. cerevisiae) | NM_001075391 | -4.898 | 5.45E-03 |
| Bt.1491.1.S1_at | *SPOCK2* | sparc/osteonectin, cwcv and kazal-like domains proteoglycan (testican) 2 | NM_001101115 | 3.051 | 1.32E-02 |
| Bt.28366.1.A1_at | *STARD10* | StAR-related lipid transfer (START) domain containing 10 | XM_592504 | 2.725 | 5.24E-03 |
| Bt.20014.1.S1_at | *STARD10* | StAR-related lipid transfer (START) domain containing 10 | XM_592504 | 2.432 | 6.57E-03 |
| Bt.28741.1.A1_at | *STEAP1* | six transmembrane epithelial antigen of the prostate 1 | XM_593128 | 2.449 | 3.34E-02 |
| Bt.9363.1.A1_at | *STIL* | SCL/TAL1 interrupting locus | XR_028515 | -3.203 | 1.20E-02 |
| Bt.13352.2.S1_at | *STRA13* | stimulated by retinoic acid 13 homolog (mouse) | NM_001114527 /// XM_870351 | -2.141 | 1.01E-02 |
| Bt.24442.1.A1_at | *SULT1A1* | sulfotransferase family, cytosolic, 1A, phenol-preferring, member 1 | NM_177521 | 2.174 | 1.07E-02 |
| Bt.12924.1.S1_at | *TACC3* | transforming, acidic coiled-coil containing protein 3 | NM_001100305 | -3.679 | 6.18E-03 |

| | | | | | |
|---|---|---|---|---|---|
| Bt.13789.1.A1_at | *TAGLN3* | transgelin 3 | NM_001034499 | -2.168 | 1.95E-02 |
| Bt.24202.1.S1_at | *TCF19* | transcription factor 19 | NM_001083684 | -3.431 | 8.60E-03 |
| Bt.8003.1.A1_at | *TCP11L2* | t-complex 11 (mouse)-like 2 | NM_001105431 | 2.187 | 4.00E-02 |
| Bt.23216.1.S1_at | *TFF2* | trefoil factor 2 | NM_001083521 | -2.884 | 6.91E-03 |
| Bt.2990.1.S1_at | *TGIF1* | TGFB-induced factor homeobox 1 | NM_001128499 /// XM_587139 | 2.187 | 2.51E-02 |
| Bt.196.1.S1_at | *TIMD4* | T-cell immunoglobulin and mucin domain containing 4 | NM_001075320 | 2.281 | 2.42E-02 |
| Bt.2899.1.S1_at | *TK1* | thymidine kinase 1, soluble | NM_001097572 | -3.215 | 1.95E-02 |
| Bt.8957.1.S1_at | *TLR7* | toll-like receptor 7 | NM_001033761 | 2.230 | 5.16E-03 |
| Bt.2551.1.S1_at | *TMEM150C* | transmembrane protein 150C | NM_001078001 | 2.327 | 1.14E-02 |
| Bt.24687.1.A1_at | *TMEM150C* | transmembrane protein 150C | NM_001078001 | 2.092 | 8.91E-03 |
| Bt.16786.1.A1_at | *TMEM156* | transmembrane protein 156 | NM_001083469 | 2.349 | 5.24E-03 |
| Bt.5573.1.S1_at | *TMEM88* | transmembrane protein 88 | NM_001098378 | -2.409 | 4.91E-03 |

| | | | | | |
|---|---|---|---|---|---|
| Bt.2899.1.S2_at | *TOP2A* | topoisomerase (DNA) II alpha 170kDa | XM_001254457 | -4.452 | 5.99E-03 |
| Bt.5362.2.S1_a_at | *TOP2A* | topoisomerase (DNA) II alpha 170kDa | XM_001254457 | -5.446 | 4.83E-03 |
| Bt.22265.1.S1_at | *TPX2* | TPX2, microtubule-associated, homolog (Xenopus laevis) | NM_001098898 | -3.787 | 5.49E-03 |
| Bt.9777.1.S1_at | *TPX2* | TPX2, microtubule-associated, homolog (Xenopus laevis) | NM_001098898 | -3.890 | 6.27E-03 |
| Bt.19322.1.A1_at | *TRIP13* | thyroid hormone receptor interactor 13 | XR_027559 | -3.769 | 1.92E-02 |
| Bt.16058.1.A1_at | *TROAP* | trophinin associated protein (tastin) | NM_001024477 | -2.802 | 4.55E-03 |
| Bt.4802.1.S1_at | *TUBA1A /// TUBA1B /// TUBA1C* | tubulin, alpha 1a /// tubulin, alpha 1b /// tubulin, alpha 1c | NM_001034204 /// NM_001114856 /// NM_001166505 /// XM_001790546 /// XM_876383 | -2.326 | 1.74E-02 |
| Bt.24966.1.S1_at | *TYMS* | thymidylate synthetase | NM_001037816 | -2.366 | 1.70E-02 |
| Bt.1048.1.S1_at | *TYROBP* | TYRO protein tyrosine kinase binding protein | NM_174627 | 3.474 | 5.16E-03 |
| Bt.15980.1.A1_at | *UBE2C* | ubiquitin-conjugating enzyme E2C | NM_001037449 | -6.077 | 4.91E-03 |
| Bt.15720.1.S1_at | *UBE2L6* | ubiquitin-conjugating enzyme E2L 6 | NM_001098917 | 2.702 | 4.55E-03 |

| | | | | | |
|---|---|---|---|---|---|
| Bt.5934.1.S1_at | *UBE2L6* | ubiquitin-conjugating enzyme E2L 6 | NM_001098917 | 2.515 | 4.53E-03 |
| Bt.29203.1.A1_at | *UBE2S* | ubiquitin-conjugating enzyme E2S | NM_001076472 | -2.437 | 7.61E-03 |
| Bt.25661.1.A1_at | *UHRF1* | ubiquitin-like with PHD and ring finger domains 1 | NM_001103098 | -4.697 | 1.04E-02 |
| Bt.21513.1.A1_at | *UHRF1* | ubiquitin-like with PHD and ring finger domains 1 | NM_001103098 | -7.020 | 7.17E-03 |
| Bt.5578.1.S1_at | *VCAM1* | vascular cell adhesion molecule 1 | NM_174484 | 2.527 | 1.57E-02 |
| Bt.15925.1.S1_at | *VEGFA* | vascular endothelial growth factor A | NM_174216 | -2.717 | 8.94E-03 |
| Bt.28878.1.S1_at | *VNN1* | vanin 1 | NM_001024556 | 2.886 | 4.85E-02 |
| Bt.19567.1.A1_at | *VRK1* | vaccinia related kinase 1 | NM_001038224 | -2.749 | 1.77E-02 |
| Bt.20606.2.S1_at | *VSIG4* | V-set and immunoglobulin domain containing 4 | NM_001046529 | 2.889 | 1.54E-02 |
| Bt.11159.1.S1_at | *WDFY4* | WDFY family member 4 | XM_606192 | 2.164 | 4.55E-03 |
| Bt.13336.1.A1_at | *WIPF3* | WAS/WASL interacting protein family, member 3 | XM_001254241 | 2.207 | 5.81E-03 |
| Bt.29462.1.S1_at | *YPEL3* | yippee-like 3 (Drosophila) | XR_042991 | 2.830 | 1.56E-02 |

| | | | | | |
|---|---|---|---|---|---|
| Bt.17157.1.A1_at | *ZFP36* | zinc finger protein 36, C3H type, homolog (mouse) | NM_174493 | 2.269 | 6.90E-03 |
| Bt.4441.1.S1_at | *ZSWIM7* | zinc finger, SWIM-type containing 7 | XM_591893 | 2.282 | 1.30E-02 |
| Bt.27172.1.A1_at | *ZWINT* | ZW10 interactor | NM_001040529 | -3.083 | 1.21E-02 |
| Bt.10855.1.S1_at | --- | --- | --- | 4.942 | 1.08E-02 |
| Bt.22026.1.S1_at | --- | --- | --- | 4.608 | 9.10E-03 |
| Bt.22366.1.S1_at | --- | --- | --- | 3.541 | 1.22E-02 |
| Bt.1370.1.S1_at | --- | --- | --- | 3.462 | 7.41E-03 |
| Bt.21238.1.S1_at | --- | --- | --- | 3.307 | 4.98E-03 |
| Bt.7331.1.S2_at | --- | --- | --- | 3.170 | 1.50E-02 |
| Bt.24247.1.S1_at | --- | --- | --- | 3.151 | 4.53E-03 |
| Bt.11587.3.A1_a_at | --- | --- | --- | 3.088 | 6.32E-03 |
| Bt.12500.2.S1_a_at | --- | --- | --- | 3.083 | 1.79E-02 |

| | | | | | |
|---|---|---|---|---|---|
| Bt.26896.1.S1_at | --- | --- | --- | 3.058 | 1.41E-02 |
| Bt.22969.1.S1_a_at | --- | --- | --- | 2.948 | 5.79E-03 |
| Bt.26521.1.S1_at | --- | --- | --- | 2.936 | 2.07E-02 |
| Bt.26636.1.S1_at | --- | --- | --- | 2.924 | 5.74E-03 |
| Bt.6556.1.S1_at | --- | --- | --- | 2.884 | 2.24E-02 |
| Bt.1537.1.S1_at | --- | --- | --- | 2.827 | 6.83E-03 |
| Bt.22491.1.A1_at | --- | --- | --- | 2.791 | 1.71E-02 |
| Bt.18081.2.S1_at | --- | --- | --- | 2.784 | 1.76E-02 |
| Bt.7033.1.S1_at | --- | --- | --- | 2.752 | 6.92E-03 |
| Bt.12619.1.S1_at | --- | --- | --- | 2.732 | 9.70E-03 |
| Bt.8947.1.S1_at | --- | --- | --- | 2.695 | 1.94E-02 |
| Bt.26722.1.A1_a_at | --- | --- | --- | 2.676 | 1.12E-02 |

| | | | | | |
|---|---|---|---|---|---|
| Bt.12718.1.A1_at | --- | --- | --- | 2.670 | 8.51E-03 |
| Bt.13573.1.A1_at | --- | --- | --- | 2.629 | 6.74E-03 |
| Bt.13214.1.S1_at | --- | --- | --- | 2.581 | 1.95E-02 |
| Bt.11271.1.S1_at | --- | --- | --- | 2.564 | 1.26E-02 |
| Bt.5262.1.S1_at | --- | --- | --- | 2.560 | 2.03E-02 |
| Bt.24872.1.S1_at | --- | --- | --- | 2.508 | 7.81E-03 |
| Bt.15730.2.S1_at | --- | --- | --- | 2.469 | 5.74E-03 |
| Bt.12550.1.S1_at | --- | --- | --- | 2.458 | 4.06E-02 |
| Bt.1858.1.A1_at | --- | --- | --- | 2.420 | 2.85E-02 |
| Bt.20164.1.S1_at | --- | --- | --- | 2.403 | 8.26E-03 |
| Bt.5970.1.S1_a_at | --- | --- | --- | 2.372 | 5.79E-03 |
| Bt.24461.1.A1_at | --- | --- | --- | 2.369 | 1.95E-02 |

| | | | | | |
|---|---|---|---|---|---|
| Bt.1518.1.S1_at | --- | --- | --- | 2.368 | 1.53E-02 |
| Bt.23278.1.S1_at | --- | --- | --- | 2.364 | 4.91E-03 |
| Bt.10499.1.S1_at | --- | --- | --- | 2.353 | 1.27E-02 |
| Bt.8308.2.S1_at | --- | --- | --- | 2.353 | 1.21E-02 |
| Bt.11001.1.S1_at | --- | --- | --- | 2.344 | 9.03E-03 |
| Bt.28346.1.A1_at | --- | --- | --- | 2.332 | 1.83E-02 |
| Bt.8743.1.S1_at | --- | --- | --- | 2.319 | 4.66E-03 |
| Bt.8308.1.A1_at | --- | --- | --- | 2.319 | 1.64E-02 |
| Bt.4285.1.S1_at | --- | --- | --- | 2.314 | 8.58E-03 |
| Bt.21576.1.S1_at | --- | --- | --- | 2.300 | 5.16E-03 |
| Bt.4110.1.S1_at | --- | --- | --- | 2.293 | 2.26E-02 |
| Bt.10092.1.S1_at | --- | --- | --- | 2.293 | 1.27E-02 |

| | | | | | |
|---|---|---|---|---|---|
| Bt.23597.1.S1_at | --- | --- | --- | 2.264 | 6.57E-03 |
| Bt.13975.1.S1_a_at | --- | --- | --- | 2.243 | 1.25E-02 |
| Bt.7603.1.S1_at | --- | --- | --- | 2.200 | 4.91E-03 |
| Bt.22390.3.A1_at | --- | --- | --- | 2.194 | 1.39E-02 |
| Bt.9101.1.S1_at | --- | --- | --- | 2.194 | 5.16E-03 |
| Bt.2810.1.A1_at | --- | --- | --- | 2.186 | 1.66E-02 |
| Bt.16141.1.S1_at | --- | --- | --- | 2.177 | 8.51E-03 |
| Bt.2629.1.S1_at | --- | --- | --- | 2.176 | 4.70E-02 |
| Bt.25305.1.A1_at | --- | --- | --- | 2.147 | 4.55E-03 |
| Bt.19267.1.A1_at | --- | --- | --- | 2.139 | 4.69E-02 |
| Bt.20308.1.S1_at | --- | --- | --- | 2.123 | 3.56E-02 |
| Bt.4001.1.S1_at | --- | --- | --- | 2.113 | 4.47E-02 |

| | | | | | |
|---|---|---|---|---|---|
| Bt.23514.1.S1_at | --- | --- | --- | 2.097 | 6.65E-03 |
| Bt.21047.1.A1_at | --- | --- | --- | 2.094 | 2.11E-02 |
| Bt.24760.1.S1_at | --- | --- | --- | 2.084 | 5.16E-03 |
| Bt.27356.1.S1_at | --- | --- | --- | 2.061 | 5.24E-03 |
| Bt.22379.1.S1_at | --- | --- | --- | 2.058 | 1.58E-02 |
| Bt.14576.1.A1_at | --- | --- | --- | 2.054 | 5.88E-03 |
| Bt.28439.1.S1_a_at | --- | --- | --- | 2.053 | 1.31E-02 |
| Bt.27328.1.S1_at | --- | --- | --- | 2.045 | 1.01E-02 |
| Bt.9662.1.S1_at | --- | --- | --- | 2.038 | 1.91E-02 |
| Bt.14379.1.A1_at | --- | --- | --- | 2.037 | 1.47E-02 |
| Bt.19757.1.A1_at | --- | --- | --- | 2.023 | 8.51E-03 |
| Bt.5392.1.S1_at | --- | --- | --- | 2.015 | 1.54E-02 |

| | | | | | |
|---|---|---|---|---|---|
| Bt.12986.1.S1_at | --- | --- | --- | 2.011 | 1.31E-02 |
| Bt.14166.1.A1_at | --- | --- | --- | 2.011 | 9.49E-03 |
| Bt.29271.1.A1_at | --- | --- | --- | 2.007 | 5.53E-03 |
| Bt.24927.1.A1_at | --- | --- | --- | 2.006 | 9.95E-03 |
| Bt.13588.3.A1_at | --- | --- | --- | 2.001 | 2.77E-02 |
| Bt.22314.1.S1_at | --- | --- | --- | -2.021 | 7.41E-03 |
| Bt.14026.1.A1_at | --- | --- | --- | -2.024 | 1.08E-02 |
| Bt.1130.1.S1_at | --- | --- | --- | -2.036 | 6.74E-03 |
| Bt.27997.1.S1_at | --- | --- | --- | -2.056 | 6.44E-03 |
| Bt.11993.1.S1_at | --- | --- | --- | -2.057 | 2.00E-02 |
| Bt.12576.3.S1_a_at | --- | --- | --- | -2.081 | 5.26E-03 |
| Bt.6133.2.A1_at | --- | --- | --- | -2.090 | 1.85E-02 |

| | | | | | |
|---|---|---|---|---|---|
| Bt.12610.1.A1_at | --- | --- | --- | -2.096 | 3.77E-02 |
| Bt.21820.1.S1_at | --- | --- | --- | -2.127 | 2.30E-02 |
| Bt.6404.1.S1_at | --- | --- | --- | -2.161 | 1.51E-02 |
| Bt.9202.1.S1_at | --- | --- | --- | -2.171 | 1.14E-02 |
| Bt.29327.1.A1_at | --- | --- | --- | -2.181 | 1.44E-02 |
| Bt.137.1.S1_at | --- | --- | --- | -2.235 | 4.91E-03 |
| Bt.13954.1.S1_at | --- | --- | --- | -2.272 | 2.54E-02 |
| Bt.17377.1.S1_at | --- | --- | --- | -2.300 | 1.27E-02 |
| Bt.12063.1.S1_at | --- | --- | --- | -2.352 | 2.62E-02 |
| Bt.24218.2.S1_a_at | --- | --- | --- | -2.377 | 7.14E-03 |
| Bt.287.1.S1_at | --- | --- | --- | -2.512 | 4.53E-03 |
| Bt.23854.1.A1_at | --- | --- | --- | -2.610 | 7.87E-03 |

| | | | | | |
|---|---|---|---|---|---|
| Bt.9841.1.S1_at | --- | --- | --- | -2.757 | 6.81E-03 |
| Bt.9577.1.S1_a_at | --- | --- | --- | -2.890 | 7.58E-03 |
| Bt.20890.1.S1_at | --- | --- | --- | -3.257 | 6.74E-03 |
| Bt.9455.1.A1_at | --- | --- | --- | -3.607 | 5.24E-03 |
| Bt.24288.1.A1_at | --- | --- | --- | -3.667 | 6.57E-03 |
| Bt.26530.1.S1_at | --- | --- | --- | -3.724 | 5.30E-03 |
| Bt.16925.1.A1_at | --- | --- | --- | -4.071 | 5.30E-03 |
| Bt.25837.1.S1_at | --- | --- | --- | -4.201 | 9.31E-03 |
| Bt.23271.1.A1_at | --- | --- | --- | -4.345 | 4.83E-03 |
| Bt.10648.1.S1_at | --- | --- | --- | -4.461 | 5.24E-03 |
| Bt.2725.1.S1_at | --- | --- | --- | -4.564 | 6.27E-03 |
| Bt.2.1.S1_at | --- | --- | --- | -5.436 | 6.57E-03 |

| | | | | | |
|---|---|---|---|---|---|
| Bt.23658.1.A1_at | --- | --- | --- | -5.989 | 5.06E-03 |
| Bt.4138.2.S1_at | --- | --- | --- | -6.109 | 4.53E-03 |
| Bt.24827.1.A1_at | --- | --- | --- | -7.608 | 8.58E-03 |

Analysis by ANOVA in Partek, with > 2-fold change, $P < 0.05$ (n = 543), in alphabetical order. Probe sets which do not have gene assignations were placed at the end of the list. The *P*-value for multiple corrections was determined by the step-up FDR method.
